# The Paradox of Music-Evoked Sadness: An Online Survey

**DOI:** 10.1371/journal.pone.0110490

**Published:** 2014-10-20

**Authors:** Liila Taruffi, Stefan Koelsch

**Affiliations:** Department of Educational Sciences & Psychology and Cluster of Excellence, “Languages of Emotion,” Freie Universität Berlin, Berlin, Germany; The University of Chicago, United States of America

## Abstract

This study explores listeners’ experience of music-evoked sadness. Sadness is typically assumed to be undesirable and is therefore usually avoided in everyday life. Yet the question remains: Why do people seek and appreciate sadness in music? We present findings from an online survey with both Western and Eastern participants (N = 772). The survey investigates the rewarding aspects of music-evoked sadness, as well as the relative contribution of listener characteristics and situational factors to the appreciation of sad music. The survey also examines the different principles through which sadness is evoked by music, and their interaction with personality traits. Results show 4 different rewards of music-evoked sadness: reward of imagination, emotion regulation, empathy, and no “real-life” implications. Moreover, appreciation of sad music follows a mood-congruent fashion and is greater among individuals with high empathy and low emotional stability. Surprisingly, nostalgia rather than sadness is the most frequent emotion evoked by sad music. Correspondingly, memory was rated as the most important principle through which sadness is evoked. Finally, the trait empathy contributes to the evocation of sadness via contagion, appraisal, and by engaging social functions. The present findings indicate that emotional responses to sad music are multifaceted, are modulated by empathy, and are linked with a multidimensional experience of pleasure. These results were corroborated by a follow-up survey on happy music, which indicated differences between the emotional experiences resulting from listening to sad versus happy music. This is the first comprehensive survey of music-evoked sadness, revealing that listening to sad music can lead to beneficial emotional effects such as regulation of negative emotion and mood as well as consolation. Such beneficial emotional effects constitute the prime motivations for engaging with sad music in everyday life.

## Introduction

### “Everyday” sadness and the paradox of music-evoked sadness

Transient sadness is a so-called basic emotion that can be observed in people, independent of cultural background [1]. Sadness is characterized by low physiological and physical activity, tiredness, decreased interest in the outer world, low mood, rumination, decreased linguistic communication, and a withdrawal from social settings [2]–[5]. Moreover, sadness is particularly associated with the awareness of an irrevocable separation, the loss of an attachment figure or of a valued aspect of the self, as well as the breaking of social bonds [6]–[10]. Thus, the experience of sadness is typically assumed to be undesirable and is therefore usually avoided in everyday life. Hence, the question arises as to why people seek and appreciate sadness in music. The appeal of sad music has always been a crucial issue in aesthetics from ancient [11] to modern times [12]–[15]. It is remarkable that, despite so much philosophical debate, there is still broad disagreement about this fundamental aspect of the aesthetic experience. Nevertheless, both the scientific and the philosophical literature have consistently reported that, in addition to sadness, sad music also elicits pleasurable emotions, ranging from a sense of relief to a state of profound beauty [13], [16], [17]. However, it is difficult to draw conclusions concerning the nature of the pleasure experienced in response to sad music (e.g., whether this is due to the appraisal of musical and acoustic features or to other types of cognitive or emotional processes) due to the lack of empirical research on the topic. The study of the relationship between sadness and pleasure has been largely neglected by psychological research on music and emotion, which predominantly has focused on other aspects of the problem such as the role of individual differences [18]–[20] or the relationship between felt and perceived emotion in response to sad music [17].

### Pleasurable experiences underlying music-evoked sadness

If music-evoked sadness is, at least for some individuals, a rewarding or valuable experience, what, then, are the rewarding aspects of such an experience? In the field of philosophy, Levinson [14] investigated this problem, suggesting reward as a key concept to explain the attraction to negative emotions, and in particular to sadness, in music. He proposed that eight types of reward contribute to the appreciation of music-evoked sadness. Two are external contributions: The first, *apprehending expression*, is linked to the observation that negatively valenced responses to music facilitate our grasp of the expression in a musical work [21]. The second consists of the Aristotelian theory of *catharsis* [11] applied to the musical domain. According to this theory, the negative emotional tone of sad music offers listeners the possibility of a controlled purification from a certain amount of a negative emotion afflicting them. The other six rewards are divided into two groups. The first group includes the following rewards: *savoring feeling* (i.e., savoring the qualitative aspects of sadness for its own sake); *understanding feeling* (i.e., sadness is perceived and appraised more clearly); and *emotional assurance* (i.e., music-evoked sadness allows listeners to reassure themselves about their ability to feel intense emotions). These rewards share the characteristic of being detached from contextual implications. This means that music-evoked sadness is not directed at any extra-musical (“real-life”) circumstance that could evoke sadness, and, therefore, is deprived of its aversive aspects (e.g., grieving due to the loss of a loved one). The second group includes three other rewards: *emotional resolution* (i.e., a sense of mastery and control listeners derive from identifying themselves with sad music resolving happily); *expressive potency* (i.e., identifying with the music to the point of imagining oneself to have the same richness and spontaneity of the sadness expressed by music); and *emotional communion* (i.e., sharing the sadness of another human being such as the composer). These last rewards are closely connected to the ability to imagine oneself in the emotional condition portrayed by the music. According to Levinson’s theory, imagination and freedom from “real-life” implications mediate the passage from music-evoked sadness to music-evoked reward. However, such rewards have not yet been empirically investigated.

Preliminary evidence for the rewards of music-evoked sadness can be found in studies which showed that pleasant emotions, such as blitheness and wonder, are elicited in response to sad music [17], [20], [22], [23]. Because it is well established that pleasure refers to the subjective hedonic component of reward [24], [25], the pleasant emotions evoked in these studies may be, for instance, the outcome of any combination of the above-mentioned rewards of music-evoked sadness.

In addition to Levinson’s theory, Panksepp [26] found that sad music is more effective for arousing “chills” (i.e., intensely pleasurable responses to music) than happy music. Consequently, he argued that the neural substrate of social loss might entail similar neurochemicals (e.g., oxytocin or opioids) involved also in the “chill” response [26], [27]. Furthermore, Huron proposed that the pleasure experienced through sad music is due to the consoling effects of prolactin, a hormone usually released when people are sad or weeping [28]. However, no direct evidence is yet available for a role of prolactin, and there is a lack of empirical data supporting any of the proposed theories [14], [26], [28].

### Situational factors and listener characteristics modulating the appreciation of sad music

Although the existing literature reports a wide range of responses to sad music, varying from “like” to “dislike” or even “hate” [18], [20], [28], very little is known about which factors modulate the appreciation of sad music. Nevertheless, situational factors and listener characteristics might explain a significant portion of the variance in the emotional and aesthetic responses evoked by sad music.

With regard to situational factors, music-evoked emotions are strongly influenced by the situational conditions of exposure to music [29]–[31], as well as by the purpose that music serves in a given situation [32]. Thus, the investigation of the situational factors underlying engaging with sad music is important to understand why sad music is appreciated (if individuals actively choose to listen to sad music, then we can assume that they appreciate such music). Although no direct evidence has been published concerning the situations in which people engage with sad music, two qualitative studies identified a number of explicit functions achieved by listening to sad music, such as *re-experiencing affect*, *cognitive*, *social*, *retrieving memories*, *friend*, *distraction*, and *mood enhancement* [33], [34]. However, because these studies are limited by their small sample sizes (for example, only five participants were recruited by Garrido and Schubert [33]), further research should extend their findings to a broader population.

With regard to the listener characteristics, individual differences in personality traits can help to clarify why some listeners strongly appreciate sad music while others avoid it. Surprisingly, relatively few studies have specifically assessed the contribution of personality to the inclination of listening to sad music [18]–[20]. Vuoskoski and colleagues [20] discovered that *openness to experience,* global *empathy* and its subscales, *fantasy,* and *empathic concern* significantly correlate with liking of sad music and intensity of emotional responses evoked by sad music. Moreover, Vuoskoski and Eerola [19] found that global *empathy* and its subscales, *fantasy,* and *empathic concern* contribute to sadness evoked by unfamiliar music, while only *fantasy* plays a role in the case of familiar music. On the other hand, Garrido and Schubert [18] showed that *absorption* and *musical empathy* predict enjoyment of negative emotions in response to music. Thus, although more empathic individuals seem to appreciate sad music more than less empathic individuals, further studies could help to specify the nature of the association between the trait empathy and the appreciation of sad music (e.g., whether it is due to a specific use of sad music in more empathic individuals). Another factor representing a good candidate for modulating the appreciation of sad music is mood. Moods are affective states lower in intensity and longer in duration than emotions, and usually not directed at any specific object [35]. A number of studies reported mood-congruent effects on liking of sad music [36], [37]. For example, Schellenberg and colleagues [37] statistically eliminated the typical preference for happy music over sad music after a demanding distractor task (which aimed to induce a negative mood in the participants). Moreover, Hunter and colleagues [36] were able to attribute this effect to sad mood, by showing that liking of sad music increases when listeners are in a sad mood.

### Principles underlying the evocation of sadness by music

Emotions can be evoked by music in different ways. Several researchers [38]–[40] theoretically introduced a number of principles through which music listening may evoke emotions. The principles underlying emotional responses to music encompass e.g., *appraisal*, *evaluative conditioning*, *contagion*, *memory*, *expectancy*, *imagination or visual imagery*, *understanding*, *rhythmic entrainment,* and *social functions* [39], [40]. To date, no evidence has been published indicating the most relevant principles through which sadness is usually evoked. Moreover, it still needs to be established whether different personality types contribute to elicit sadness through specific principles.

### The present study

The aim of this study was to provide a better understanding of why people engage with sad music. We collected responses from a large multi-ethnic sample of participants, covering diverse age groups, through means of an online survey. In particular, we focused on the rewarding aspects of music-evoked sadness suggested by Levinson [14], as well as the relative contribution of listener characteristics (e.g., mood and personality) and situational factors to the appreciation of sad music. Because our knowledge of how many listeners experience sadness in response to sad music is largely based on very limited data, and because emotions other than sadness can also be elicited by sad music [17], [20], another purpose was to identify the most frequent emotions experienced in response to sad music. Furthermore, we also examined the role of the above-mentioned principles [39], [40] in evoking sadness as well as their interaction with personality traits. Because we obtained responses from a multi-ethnic sample of participants, we further compared the Western and Eastern participants’ responses to investigate whether broad cultural differences influence the reward and/or the emotional experiences associated with listening to sad music. Finally, to further discriminate which uses and rewards are specific to sad music compared to other types of music (e.g., happy music), we distributed a second survey on happy music to another sample of participants.

## Methods

### Ethics statement

The present study was an online survey which was completely voluntary and anonymous, i.e. no personal data were collected except age, gender, and nationality. Moreover, no financial compensation was provided. We obtained informed consent from all participants through an online form with which the survey started. However, it was not possible to obtain an additional informed consent from the guardians on behalf of the under-aged subjects (6 subjects with minimum age 16). Obtaining such an informed consent from a guardian wouldn’t have been possible, given that the survey was completely anonymous. Informing under-aged participants that they could not take part, would still have left the opportunity that they might take part without their guardians approval by entering an incorrect (older) age. These issues can thus be considered as a general problem that applies to web-based anonymous surveys. Nevertheless, we made sure that the survey did not feature any material/question that could have posed any negative influence or risk on minors. In addition, participants were informed that they could withdraw at any time without giving a reason and without any negative consequence. Participants were recruited through electronic mailing lists of students and through the newsletter of the Cluster Languages of Emotion. The study was conducted according to the Declaration of Helsinki and approved by the ethics committee of the Psychology Department of the Freie Universität Berlin. Because in the ethics application nothing was stated about the age of participants, we therefore assume that the ethics committee did not see anything critical about the questions being answered by minors.

### Participants and data collection procedure

Data were obtained from 772 individuals: 495 females (64.1%) aged 16–78 years (M = 28.3, SD = 9.0) and 277 males (35.9%) aged 16–68 years (M = 28.6, SD = 8.1). 408 participants grew up in Europe (52.8%, 266 females), 224 (29.1%, 128 females) in Asia, 122 (15.8%, 88 females) in North America, 10 (1.3%, 7 females) in South America, 6 (0.8%, 5 females) in Australia, and 2 (0.2%, 1 female) in Africa. With respect to their musical training, 40 (5.2%, 31 females) respondents reported to be professional musicians, 66 (8.5%, 31 females) semi-professional musicians, 230 (29.8%, 143 females) amateur musicians, and 436 (56.5%, 290 females) non-musicians. With regard to their musical engagement, 500 (64.8%, 319 females) participants reported being music lovers, 262 (33.9%, 168 females) liking music, and 10 (1.3%, 8 females) not being music lovers.

### Materials

Data were collected using an online survey. In total, the survey featured 76 items. Participants were instructed to complete the survey individually, and in a quiet environment without listening to any music. The survey was programmed and administered online between the 3^rd^ of February and the 3^rd^ of June 2013, using the software *Unipark* (Unipark, Germany; www.unipark.info). The average duration to survey completion was 20 minutes.

The survey was divided into seven sections (further details are explained below): (1) *Core Details*; (2) *Musical Training and Musical Engagement*; (3) *Sad Music*; (4) *Principles Underlying the Evocation of Sadness by Music*; (5) *Rewarding Aspects of Music-Evoked Sadness*; (6) *Favourite Sad Music*; and (7) *Personality Questionnaires*. Items were randomised among each section.

#### Core Details, Musical Training, and Musical Engagement

The first two sections consisted of items used to obtain demographic data, details about musical training, subjective relevance of music, and preferred music genre(s).

#### Sad Music

The third section comprised items on sad music listening habits, including: frequency of listening to sad music; situation-related factors and their importance for listening to sad music; liking of sad music according to the listener’s mood (positive and negative); and emotions evoked by sad music (see Table S1 for the list of items). Participants provided quantitative ratings on 7-point Likert scales for the first three items. In addition to the ratings, the item related to the situational factors was designed as an open-ended response where participants were asked to provide one or more examples of situations in which they engage with sad music. The explanatory nature of the open response was adopted to be able to draw conclusions on the motivations for selecting sad music with regard to the situational factors. For the last item, “emotions evoked by sad music”, participants were asked to indicate the emotions that they frequently experience when listening to sad music. They could select either one or more emotions from the nine emotions listed in the Geneva Emotional Music Scale (GEMS-9) [23], or add their own alternative responses. Furthermore, participants were given the opportunity to answer that sad music does not evoke any particular emotion in them. The GEMS was selected as ideal instrument to measure the subjective emotional experience of participants, because it provides a nuanced assessment of music-evoked emotions [23], [41]. It comprises nine categories (*wonder*, *transcendence*, *tenderness*, *nostalgia*, *peacefulness*, *power*, *joyful activation*, *tension,* and *sadness*), which condense into three main factors: *sublimity*; *vitality*; and *unease*.

#### Principles Underlying the Evocation of Sadness by Music

The fourth section consisted of a 7-item questionnaire designed to evaluate the role of different principles underlying the evocation of sadness in listeners [39], [40]. However, not all of these principles were suitable to be translated into clear statements. Thus, the present study used only those which could have been reasonably operationalized by the use of self-reports (i.e., *memory*, *imagination*, *contagion*, *appraisal*, and *social functions*; see Table S2 for the list of items). Ratings were given on a 7-point Likert scale (1 = *strongly disagree* and 7 = *strongly agree*).

#### Rewarding Aspects of Music-Evoked Sadness

Participants were then presented with 13 items devised to explore possible rewarding aspects of music-evoked sadness. These items (see Table S3) were designed on the basis of Levinson’s theory on negative emotions [14] and integrated with three items indicated by previous studies on sad music and emotion regulation [28], [34], [42]–[44]. All ratings were provided on a 7-point Likert scale (1 = *strongly disagree* and 7 = *strongly agree*).

#### Favourite Sad Music

In the sixth section participants were asked to provide one or more example(s) of their favourite sad music (either instrumental or with lyrics). This question was included because respondents were provided with neither a definition of sad music nor examples of sad music in the survey, but were instead instructed to focus on “self-identified sad music” (as in Van den Tol and Edwards [34]). Given that “self-identified sad music” may represent music that does not sound “sad” to any other listener (for example, because of personal associations with the music, such as the break up of a relationship), we examined the examples of sad music provided by participants to determine whether they are consistent with the Western cultural conventions of representing sadness in music. Specifically, we made use of the tagging system supported by the online music database www.last.fm. In addition, we retrieved a number of acoustic and musical features of the instrumental pieces named by the participants (for details see the Results section).

#### Personality Questionnaires

The final part of the survey included two measures of individual differences in trait empathy and personality factors. Empathy was assessed via the Interpersonal Reactivity Index (IRI) [45]. The IRI includes 28 items, divided in four sub-scales measuring the following related aspect of emotional empathy: *fantasy*; *perspective-taking*; *empathic concern*; and *personal distress*. As a means of limiting the experimental procedure to a maximum of 20 minutes, personality traits were assessed by the Ten-Item Personality Inventory (TIPI) [46]. This is a brief version of the Big Five Inventory (BFI) [47] and it covers the following five personality domains: *extraversion*; *agreeableness*; *conscientiousness*; *emotional stability*; and *openness to experience*.

## Results

### Which are the rewarding aspects of music-evoked sadness?

A principal component analysis (PCA) with oblique rotation (direct oblimin) was carried out on the ten items describing the rewarding aspects of music-evoked sadness (in the preliminary analysis three items were excluded because of their low correlations). The Kaiser-Meyer-Olkin measure verified the sampling adequacy for the analysis, KMO = .83, and all KMO values for individual items were >.76, which is well above the acceptable limit of .5 [48]. Bartlett’s test of sphericity showed that correlations between items were sufficiently large for a PCA (χ^2^ (45) = 3402.65, *p*<.001). An initial analysis was computed to obtain eigenvalues for each dimension in the data. Four dimensions had eigenvalues over Jolliffe’s criterion of 0.7 and, in combination, explained 76.6% of the variance. Given the large sample size, and the convergence of the scree plot and Jolliffe’s criterion on four dimensions, these four dimensions were retained in the final analysis. Table 1 shows the factor loadings after rotation. The items that cluster on the same dimensions suggest to interpret dimension 1 as the *reward of imagination*, where music-evoked sadness has pleasurable effects due to the engagement of imaginative processes (e.g., “I imagine I have the same rich expressive ability as present in the music”). Dimension 2 represents the *reward of emotion regulation,* which includes statements about the rewarding effects derived from regulation of negative moods and emotions (e.g., “Experiencing sadness through music makes me feel better after listening to it, and thus has a positive impact on my emotional well-being”). Dimension 3 represents the *reward of empathy*, which includes statements about the pleasurable effects of music-evoked sadness due to mood-sharing and virtual social contact through the music (e.g., “I like to empathise with the sadness expressed in the music, as if it were another individual”). Dimension 4 represents the *reward of no “real-life” implications*, which includes statements about the pleasure listeners can take in music-evoked sadness due to its lack of contextual implications (e.g., “I can enjoy the pure feeling of sadness in a balanced fashion, neither too violent, nor as intense as in real-life”). With regard to the consistency of the extracted factors, *imagination* had high reliability (Cronbach’s α>.92) and *no “real-life” implications*, *emotion regulation* and *empathy* had good reliability (Cronbach’s α>.7).

**Table 1:** Factor loadings for explanatory factor analysis with direct oblimin rotation of the items describing the rewarding aspects of music-evoked sadness (N = 772).

	Rotated Factor Loadings			
Item	Imagination	Emotion Regulation	Empathy	No “Real- Life” Implications
Expressive Potency 1	.914			
Expressive Potency 2	.943			
Expressive Potency 3	.892			
Understanding Feelings				.805
Emotional Assurance				.844
Savoring Feeling				.581
Mood Enhancement		.927		
Catharsis		.787		
Emotional Communion			.849	
Empathic Responses			.860	
**Eigenvalues**	3.24	2.22	2.31	2.88
**% of variance**	41.57	16.85	10.44	7.75
**Α**	.92	.73	.71	.71

**Figure 1:**
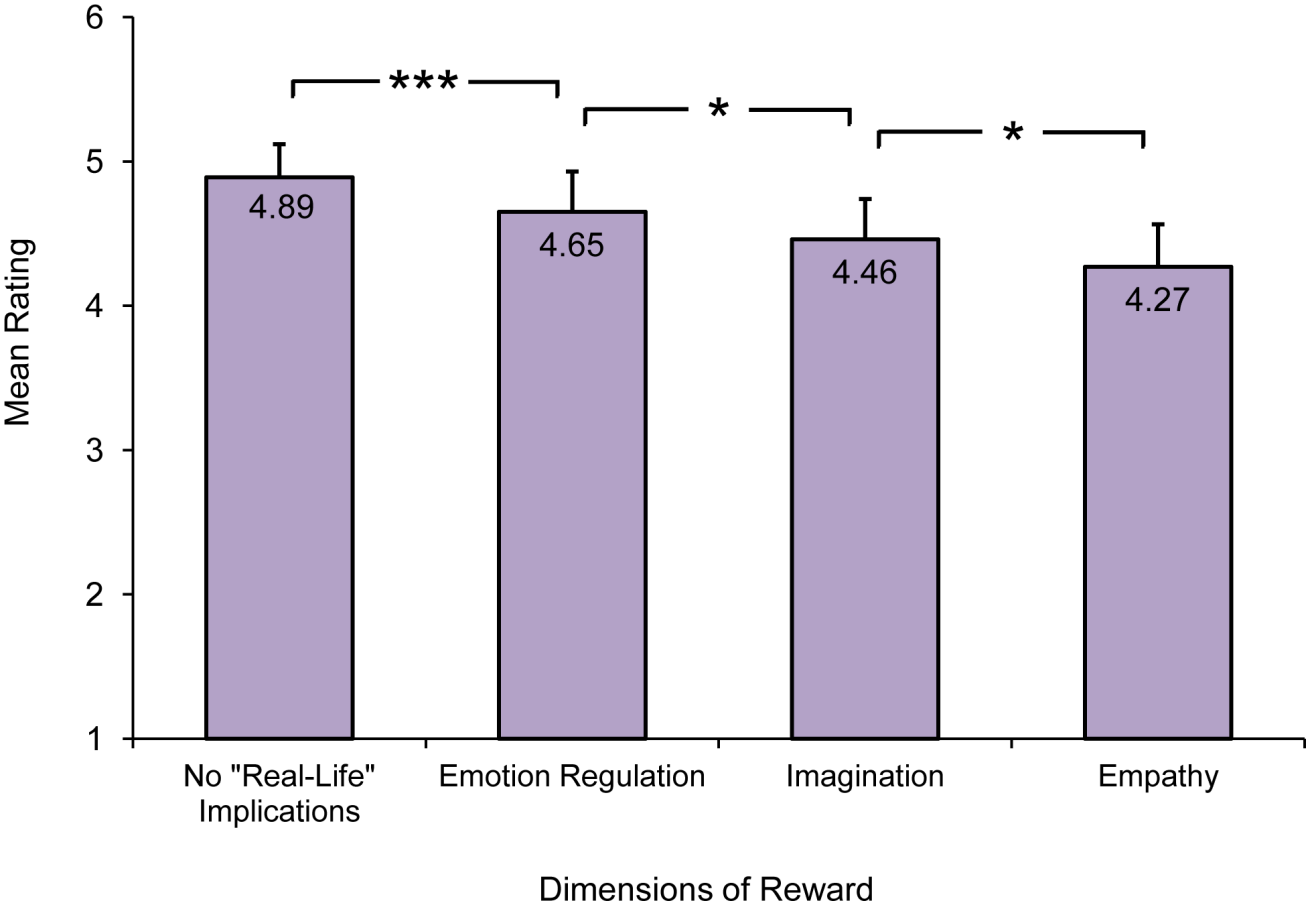
Mean ratings for each of the four dimensions of reward identified. Error bars indicate standard error of the mean, ***a *p*-level of <.001, and *a *p*-level of <.05.

A repeated-measures ANOVA, with type of reward (four levels) as within-subjects factor, was conducted to identify the most important rewards for the listeners. A significant main effect of type of reward was found, F(2.65, 2041.97) = 37.28, *p*<. 0001, *ω^2^* = .99. Bonferroni pairwise comparisons showed that there were significant differences between the mean ratings for all four factors. Figure 1 shows that *no “real-life” implications* turned out to be the most important source of reward for the listeners.

Only 31 participants (4% of all participants) took the opportunity to add their own alternative responses concerning other possible rewards that were not included in the list provided by the questionnaire. Due to the low number of responses, these answers did not provide representative information, and were therefore not further analyzed. However, to generate hypotheses for future studies, these answers are reported in the Table S4.

### In which situations do listeners engage with sad music?

Results indicate that situation-related factors play a significant role in the engagement with sad music. With regard to the following item, “How much do specific situations influence your choice to listen to sad music?”, ratings showed that situational factors are highly relevant to the choice to listen to sad music (M = 4.74, SD = 1.84, on a 7-point Likert scale ranging from 1 = *not at all* to 7 = *a lot*). 61.7% of participants (477 out of 772) provided ratings ≥5. To examine this issue further, an open-ended follow-up question asked participants to provide one or more examples of circumstances in which they engage with sad music, and to describe the function that sad music serves in those situations. A content analysis of the free responses revealed that there are several situations in which listeners engage with sad music, which are intrinsically linked to a wide spectrum of functions (i.e., emotional, social, cognitive, and aesthetic) that listening to sad music may potentially fulfill. Based on the previous literature [33], [34], the responses concerning the situational factors were grouped into the following categories (Table 2): *emotional distress*; *social*; *memory*; *relaxation and arousal*; *nature*; *musical features*; *introspection*; *background*; *fantasy*; *avoiding sad music*; *intense emotion*; *positive mood*; and *cognitive*. The category *emotional distress* includes situations in which the listeners are in a negative emotional state due to different reasons such as, for example, the loss of a loved one. In these circumstances, sad music is used as a tool for mood-enhancement (achieved, for example, through venting of negative emotion or cognitive reappraisal), consolation, or simply because it reflects the current mood. The category *social* comprises statements on social attachment and social bonding (e.g., people engage with sad music when they feel lonely or when they need to be accepted or understood), and is therefore linked to a consolatory use of sad music (achieved through mood-sharing or virtual social contact through the music). The category *memory* refers to situations in which sad music is chosen to retrieve autobiographical memories of valued past events or people. The category *relaxation and arousal* represents situations in which sad music is used as a tool to regulate arousal (e.g., quieting down before going to bed). The category *nature* refers to situations such as travelling, and being in contact with nature as well as to specific times of the day (i.e., evening) or of the year (i.e., winter). The category *musical features* is related to the aesthetic appreciation of sad music focused on the formal properties of the music, rather than on perceptions of emotional content. The categories *fantasy, cognitive*, and *introspection* represent situations in which sad music is chosen because of cognitive as well as self-related functions: Sad music is used respectively to engage creativity, to improve focus during work or while studying, and to cope with a personal problem by organizing thoughts and feelings. The category *background* refers to situations such as driving, reading or working, where sad music represents an optimal musical background. The category *avoiding sad music* comprises all the answers that stress a clear dislike for sad music, regardless of the different situational factors. The category *intense emotion* includes a number of situations in which listeners engage with sad music to experience intense emotions. Finally, the category *positive mood* includes the answers of the participants who reported to engage with sad music only when being in a positive emotional state, and, consequently, to avoid sad music when being in a negative emotional state. According to these respondents, sad music does not have any positive effect on emotional distress, but it rather contributes to perpetuate this negative affective state.

**Table 2:** Summary of the situations in which participants engage with sad music, and functions of listening to sad music in those circumstances.

Situation Category	Situation Description	Function
Emotional distress	Argument, failure, frustration, death, love-sickness or break up,need to cry, and stress	Emotional: mood enhancement (e.g., venting and cognitive reappraisal), consolation, reflection of the current mood
Social	Homesickness, feeling lonely,missing someone, need to beaccepted and understood	Social and emotional: consolation due to mood-sharing and contact
Memory	Retrieving memories of valuedpast events	Sad music as a memory trigger
Relaxation and arousal	Relaxing and getting newenergy, quieting down before goingto bed	Emotional: mood and arousal regulation
Nature	Travelling, being in contactwith nature, during specifictimes of the day (evening) orof the year (winter)	Sad music as a reflection of the environment
Musical features	Engaging with sad music not because of its emotional contentbut rather for its musical features (e.g., “sad songs are beautiful”)	Aesthetic
Introspection	Contemplating, organizing,and reappraising personal experiences	Cognitive: improve personal introspection
Background	While doing a parallelactivity such as driving, reading, working	Sad music provides a pleasant background
Fantasy	Creative thinking, lookingfor inspiration	Cognitive: engage creative thinking
Avoiding sad music	Preference for other typesof music	-
Intense emotion	Seeking a touchingemotional experience	Emotional: experience intense emotions
Positive mood	Listening to sad music only when being in a positivemood or emotional state	Emotional: mood control
Cognitive	Improving rationalthinking, obtaining a better focus	Cognitive: engage rational thinking

*Note.* Situational categories are listed in descending order according to the number of nominations.

**Figure 2:**
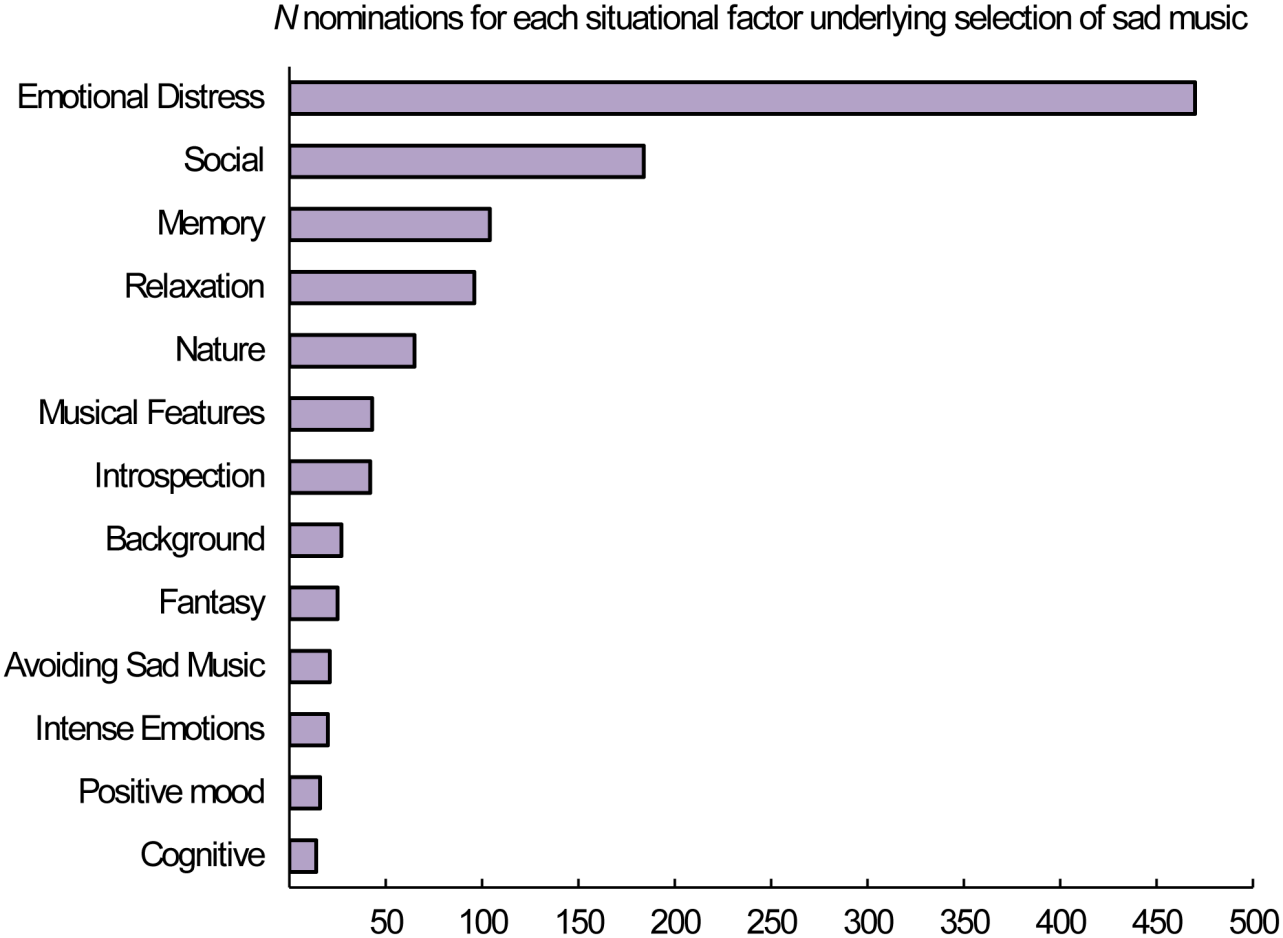
The amount of nominations for each situation-related factor underlying listening to sad music.

The number of nominations for the different situational-categories is provided in Figure 2. As can be seen, listeners reported to engage with sad music especially when experiencing emotional distress. For instance, there is a striking difference between the frequency with which participants reported the situation-related category *emotional distress* (470 nominations) and the reported frequencies of all other categories (all fewer than 184 nominations). Emotional distress is a broad concept that refers to a variety of situations. To specify this concept, we reported the most popular examples given by the participants for *emotional distress*: “when feeling sad” (109 nominations); “when experiencing lovesickness or a break up” (108); “when grieving for a loss” (51); “when experiencing stress at work/university” (48); “when feeling angry after an argument” (44); “when experiencing frustration and being disappointed with myself” (41); “when needing to release negative feelings” (29); “when feeling melancholic” (22); and “when feeling like crying” (17). Furthermore, a considerable number of participants (184 out of 772) reported engaging with sad music when they feel lonely (i.e., category *social*), whereas a small number (16) reported engaging with sad music only while experiencing a positive emotional state (i.e., category *positive mood*).

### Do mood and personality modulate the liking of sad music?

Both the engagement with, and the liking of sad music occur more frequently in a mood-congruent fashion. For instance, 54.4% of participants (420 out of 772) provided ratings ≥5 on a 7-point Likert scale (1 = *never*, 7 = *always*) in response to the statement “When I am in a sad mood I like to listen to sad music” (M = 4.44, SD = 1.80). On the other hand, 32.7% of participants (253 out of 772) provided ratings ≥5 on a 7-point Likert scale (1 = *never*, 7 = *always*) in response to the statement “When I am in a positive mood I like to listen to sad music” (M = 3.60, SD = 1.56). A paired-samples t-test revealed that the difference between the ratings for the two items is significant (*t*(771) = 10.141, *p*<.0001, *r* = .34).

A correlation analysis between the personality factors and the subscales of *empathy* with the variables of liking of sad music was performed to evaluate whether personality traits can modulate the liking of sad music. A Bonferroni correction for multiple tests was applied. Because significant correlations were weak (*r*<.2), the results (summarized in Table 3) should be interpreted with caution. However, to generate hypotheses for future studies, we also report these results. The mood-congruent liking of sad music positively correlated with global *empathy* (*r* = .114, *p*<.01) and its subscales, *fantasy* (*r* = .160, *p*<.01), and *personal distress* (*r* = .108, *p*<.01). A similar pattern was observed for the mood-incongruent liking of sad music, which positively correlated with global *empathy* (*r* = .109, *p*<.01) and its subscales, *fantasy* (*r* = .116, *p*<.01), and *perspective taking* (*r* = .142, *p*<.01), but not with *personal distress* (*r* = −.052, *p*>.05). Moreover, the mood-congruent liking of sad music negatively correlated with *emotional stability* (*r* = −.123, *p*<.01).

**Table 3:** Correlations between the mean ratings for the liking of sad music and personality traits as measured by the TIPI and IRI.

	Liking of sad music	
	Mood-congruent	Mood-incongruent
Emotional Stability	−.123**	.033
Global Empathy	.114**	.109**
Fantasy	.160**	.116**
Personal Distress	.108**	−.052
Perspective Taking	−.036	.142**

*Note.* **indicates a *p*-level of <.01.

### Which emotions are most frequently experienced in response to sad music?

**Figure 3:**
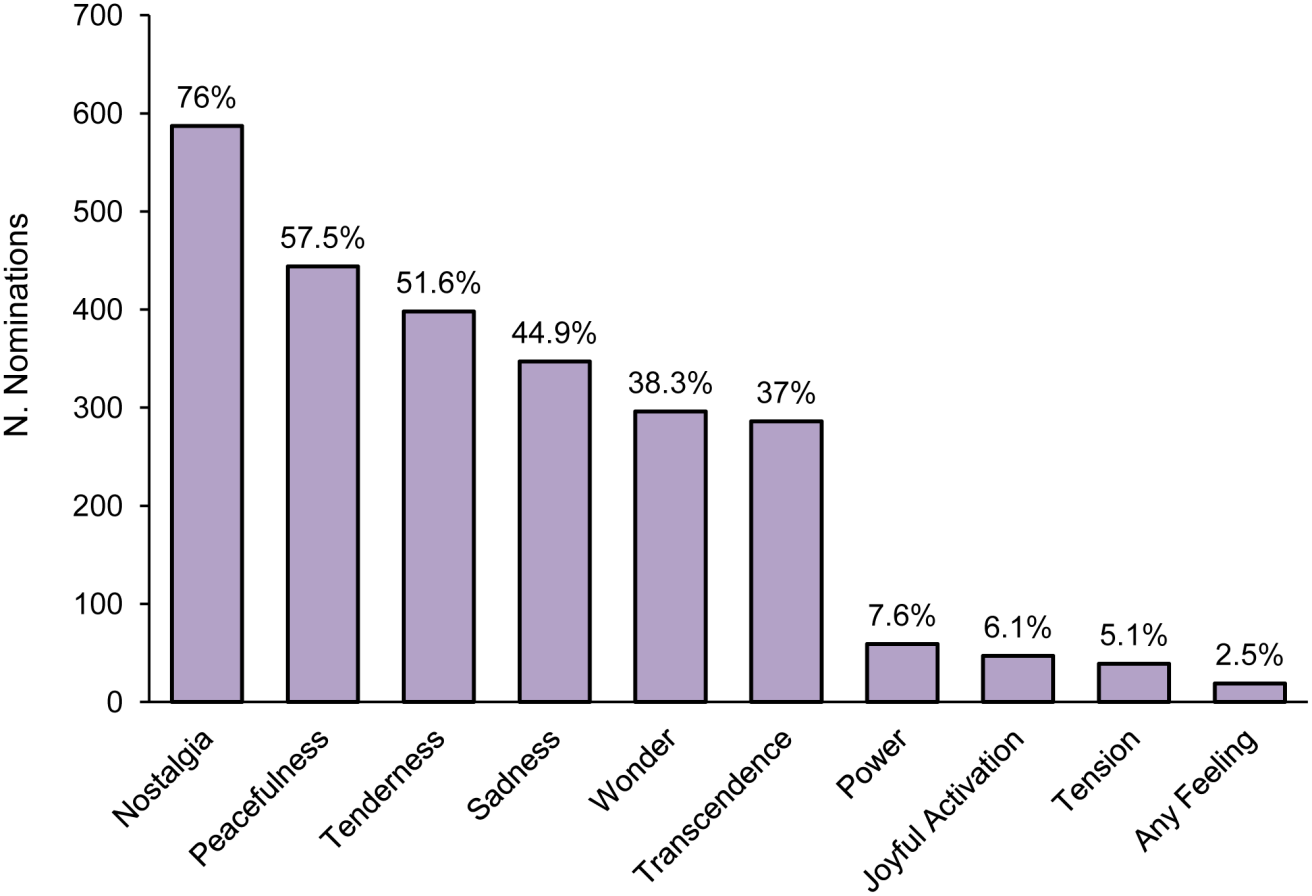
The most frequent emotions, as measured by the GEMS, evoked in response to sad music.

The survey featured an item in response to which participants indicated the most frequent emotions evoked by sad music. They could select more than one option and/or add their response alternatives (free responses are reported in Table S5). Figure 3 reports the number of nominations for each emotion. Surprisingly, nostalgia (76% of nominations), and not sadness (44.9%), was indicated as the most frequent emotion evoked by sad music. Moreover, participants also reported experiencing positive emotions, such as peacefulness (57.5%), tenderness (51.6%), and wonder (38.3%). Conversely, the percentage of nominations for joyful activation (6.1%) was low compared to the other emotions.

Interestingly, the average number of emotions that participants reported to have experienced in response to sad music (M = 3.33, SD = 1.56) correlated positively with both variables of mood-incongruent liking of sad music (*r* = .323, *p*<.01) and mood-congruent liking of sad music (*r* = .273, *p*<.01).

### What is the importance of the theoretically discussed principles underlying the evocation of sadness?

**Figure 4:**
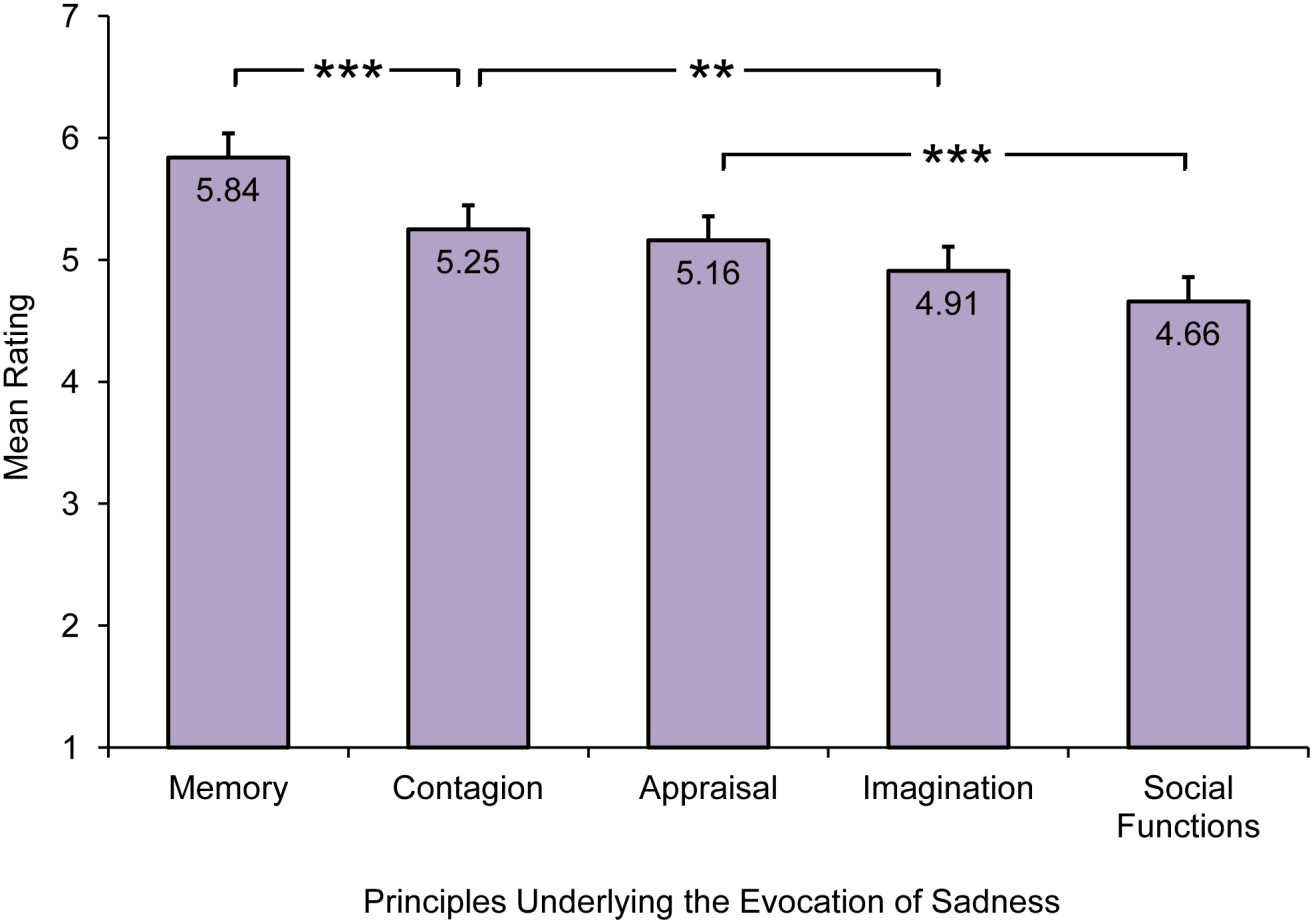
Mean ratings for each principle underlying music-evoked sadness. Error bars indicate standard error of the mean, ***a *p*-level of <.001, and **a *p*-level of <.01.

Participants who reported frequently experiencing sadness in response to sad music (N = 347) also rated to what extent they agreed/disagreed with each of the seven items (see Table S2) describing five of the principles underlying the evocation of sadness through music (i.e., *memory*, *imagination*, *contagion*, *appraisal*, and *social functions*). First, ratings were averaged across different items describing the same principle. Second, a repeated-measures ANOVA was conducted on the mean ratings for each principle, with principle type represented as a within-subjects factor, to establish which principles are most important in evoking sadness. A significant main effect of the type of principle was found, F(3.37, 1165.01) = 39.41, *p*<.0001, *ω^2^* = .22. Bonferroni pairwise comparisons showed that there were significant differences between mean ratings for all five principles, with the exception of three non-significant differences between the mean ratings given on *imagination* and *social functions*, *imagination* and *appraisal,* as well as *appraisal* and *contagion*. *Memory* was rated as the most important principle underlying the evocation of sadness. However, all the principles were judged to be relevant (all means >4.5, on a 7-point Likert scale from 1 = *strongly disagree* to 7 = *strongly agree*). Figure 4 shows the mean ratings given to each of the five principles ranked in descending order.

The data also revealed gender differences for *contagion*. On average, *contagion* was rated higher by females (M = 5.40, SE = 0.74) than by males (M = 4.92, SE = 0.14), with this difference being significant (*t*(167.12) = −3.004, *p* = .003, *r* = .24, Bonferroni-corrected).

To investigate whether differences in sadness-evocation styles might be associated with personality traits, a Pearson correlation analysis of the subscales’ scores of IRI and TIPI with the principles’ ratings was conducted. A Bonferroni correction for multiple tests was applied. The results (summarized in Table 4, only *r*>.2 are reported) reveal an association between *empathy* (total score plus all subscales) and sadness induced via *contagion* (*r* = .348, *p*<.001), via engagement in *social functions* (*r* = .399, *p*<.001), as well as via *appraisal* (*r* = .262, *p*<.001). Note that, although not reported in Table 4 (because *r<*.2), global *empathy* also significantly correlated with sadness induced via *imagination* (*r* = .184, *p*<.005) and via *memory* (*r* = .187, *p*<.001). Finally, *contagion* negatively correlated with *emotional stability* (*r* = −.296, *p*<.001).

**Table 4:** Correlations between the mean ratings for the principles underlying the evocation of sadness and personality traits as measured by the TIPI and IRI.

	Contagion	Social Functions	Appraisal
Emotional Stability	−.269**	−.054	−.072
Global Empathy	.348**	.399**	.262**
Empathic Concern	.261**	.326**	.227**
Perspective Taking	.122*	.229**	.111*
Fantasy	.309**	.319**	.242**

*Note.* Only *r*>.2 are reported. *indicates a *p*-level of <.05 and **a *p*-level of <.01.

### Are emotional responses to sad music the same across cultures?

Eastern respondents provided lower overall ratings compared to Western respondents for all items featured in the survey, with the exception of ratings for the principle of *social functions*. The following significant difference was found: Western participants reported significantly (*t*(143.49) = 3.061, *p* = .003, *r* = .25, Bonferroni-corrected) higher ratings for the principle of *memory* (M = 6.00, SE = .08) compared to Eastern participants (M = 5.41, SE = .17). According to Eastern participants, the most frequent emotion evoked in response to sad music was peacefulness (117 of 219 nominations) followed by nostalgia (115 nominations). By contrast, the ranking of these two emotions for the Western participants was reversed, with nostalgia being the most frequent emotion (451 of 530 nominations) followed by peacefulness as the second most reported emotion (316 nominations).

### Is “self-identified sad music” consistent with the cultural standards of representing sadness in music?

We made use of the tagging system supported by the online music database www.last.fm to verify that the musical pieces named by the respondents can be considered culturally valid examples of sad music. Tags are keywords or labels that listeners can use to classify music. Musical platforms, such as last.fm, are used by millions of listeners and thus offer behavioural ratings from a sufficiently large sample of user tags. We examined the tags provided for the music examples nominated by participants in the survey. First, we inspected all musical pieces nominated more than once and second, all pieces nominated only one time.

Participants reported 52 musical pieces (26 with lyrics and 26 instrumental) more than once, in a total of 165 nominations (see Table S7). Among these 52 pieces, 36 were tagged “sad” or “sadness” by last.fm users, and nine were assigned a “sadness-related” tag (e.g., “melancholic”). Three pieces received either very few or no tags and two were not found in the last.fm database. Only two musical pieces were not labeled “sad” or with a “sadness-related” tag.

A total of 380 musical pieces (233 with lyrics and 147 instrumental) were mentioned only once by the participants (see Table S8). Forty pieces were not considered in the analysis because the title provided was too general (i.e., it referred to an album rather than a song or to a symphony rather than a movement). Moreover, 71 pieces received either very few or no tags and 18 were not found in the last.fm database. Among the remaining 251 musical examples, 168 were tagged “sad” or “sadness”, and 53 were labeled with a “sadness-related” tag. Only 30 musical pieces were not assigned either a “sad” or a “sadness-related” tag.

Furthermore, through a large database of more than 30 million songs (see http://the.echonest.com/) we retrieved a number of acoustic and musical features of the instrumental pieces named by the participants, including tempo, loudness, mode, energy, dance ability, and “valence” (note that the term valence is used here to refer exclusively to the database’s musical attribute, which is derived from acoustic-driven information, not user tags; as indicated by the present data sad music can have a positive valence for listeners in certain circumstances). This was done in order to determine whether the example pieces contain musical parameters that have been consistently linked with sadness in Western music (e.g., slow tempo, low sound level, minor mode, low pitch, small intervals, legato, micro-structural irregularity, etc.; see [49]). Nominated songs with lyrics were excluded because lyrics may differ semantically from music-perceived emotion and may play a crucial role in evoking sadness [50]. Out of 142 instrumental pieces, 124 were retrievable in the database. Table 5 reports the descriptive statistics for the overall estimated tempo in BPM (M = 86.92, SD = 21.05), loudness in dB (M = −20.92, SD = 7.18), energy (M = 0.16, SD = 0.17), dance ability (M = 0.26, SD = 0.14), and valence (M = 0.13, SD = 0.14). Moreover, 52.41% of the musical pieces were written in a major mode and 47.58% in a minor mode. In addition, the energy and valence values of each piece were plotted onto a two-dimensional plane, according to the affective circumplex model of emotion [51]. Figure S1 shows that a large majority of the retrieved instrumental pieces nominated by our respondents (113 out of 124) fell into the low energy/negative valence quadrant.

**Table 5:** Descriptive statistics for the acoustic and musical features of the musical pieces nominated by the participants (N = 124).

	Minimum	Maximum	Mean	SD
Energy	0.00	0.84	0.16	0.17
Tempo (BPM)	48.21	138.35	86.92	21.05
Valence	0.00	0.67	0.13	0.14
Loudness (dB)	−39.34	−6.98	−20.92	7.18
Dance ability	0.06	0.66	0.26	0.14

*Note*. Energy measures the intensity and the powerful activity released throughout the piece. Dance ability describes whether a piece is suitable for dancing, and it combines musical elements such as tempo, rhythm stability, beat strength, and overall regularity. Valence takes into account acoustic information such as pitch, timbre, and mode. Energy, dance ability, and valence values range between 0 and 1. A value close to one indicates high energy or arousal, high dance ability, and positive valence, while a value close to zero corresponds to low energy or arousal, low dance ability, and negative valence.

### Which uses and rewards are specific to sad music?

A number of the identified uses and rewards of sad music, such as the use of music to retrieve autobiographical memories, overlap with uses and rewards of music listening in general, irrespective of the specific type of emotional experience evoked by the music [52]–[56]. To further evaluate which uses and rewards are specific to sad music, we distributed a survey on happy music to another sample of 212 participants aged 19–75 (M = 33.12, SD = 10.29; detailed demographics are reported in Table S6). The survey was a shorter version of the original one, featuring the same 25 items as used in the previous survey, divided into five sections: *Core Details*, *Musical Training*, *Happy Music*, *Rewarding Aspects of Music-Evoked Happiness*, and *Favourite Happy Musi*c. The sections *Musical Engagement*, *Principles Underlying the Evocation of Happiness by Music*, and *Personality Questionnaires* were omitted to focus this survey on the question of uses and rewards of happy music and to maintain the completion time below five minutes. The survey was distributed between the 22^nd^ of June and the 25^th^ of July 2014.

**Figure 5:**
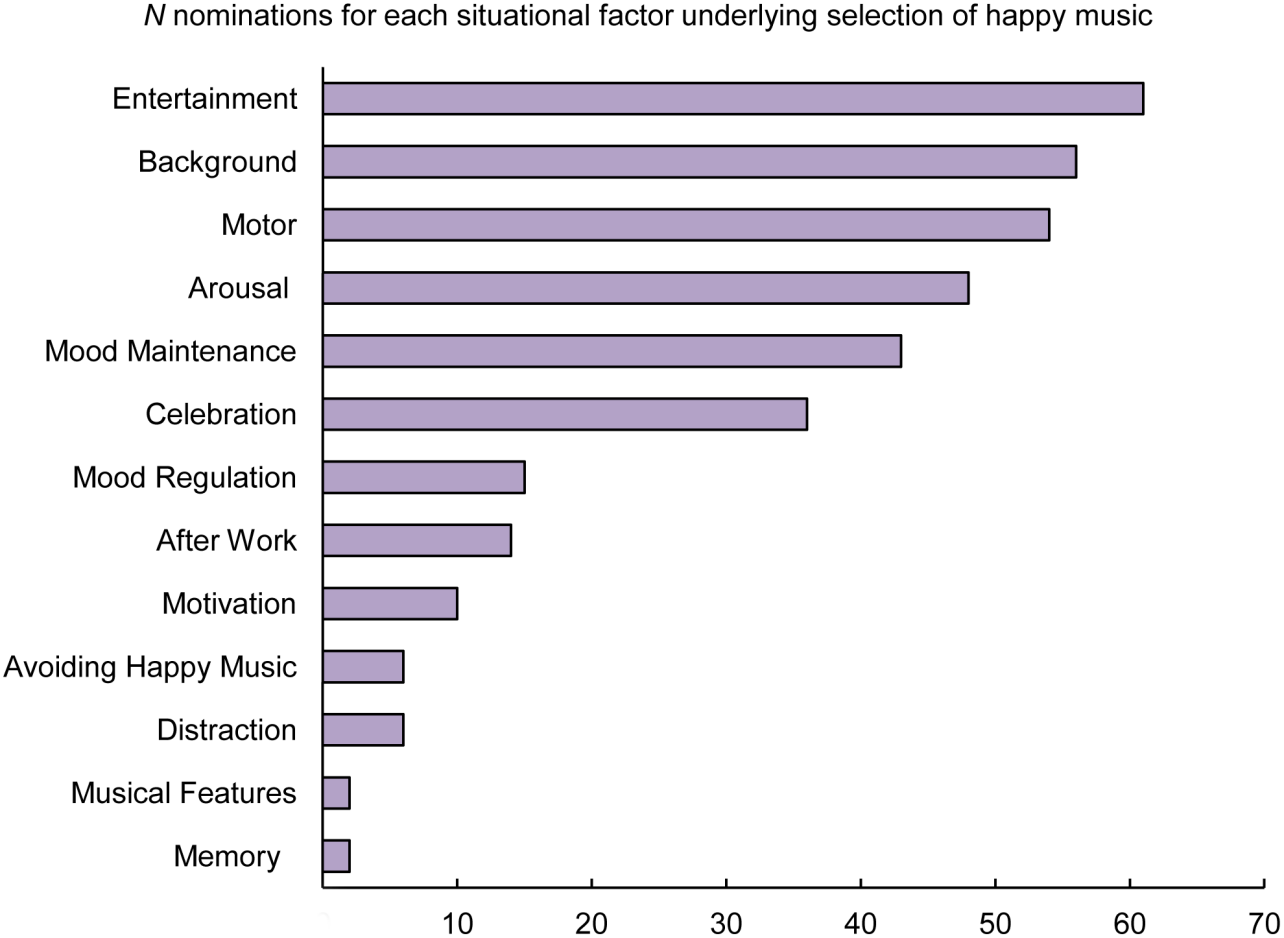
The amount of nominations for each situation-related factor underlying listening to happy music.

Participants were first asked to report in which situations they commonly engage with happy music and why. A content analysis of the free responses revealed the following situation-related categories (Table 6): *entertainment*; *background*; *motor*; *arousal*; *mood maintenance*; *celebration*; *mood regulation*; *after work*; *motivation*; *distraction*; *avoiding happy music*; *memory*; *musical features*. The category *entertainment* includes situations such as a gathering with friends or a social occasion, in which happy music is used to entertain and to create a pleasant atmosphere. The category *background* refers to situations such as travelling, driving, housekeeping, and working, in which happy music provides an optimal auditory background to a primary activity. The category *motor* represents situations such as running, dancing or working out, in which the beat is used to enhance a particular motor response. The category *arousal* includes a number of situations in which happy music is used as a tool to regulate arousal, such as energizing in the morning, releasing energy or simply relaxing. The categories *mood maintenance, mood regulation*, and *distraction* represent situations in which happy music is selected to achieve various emotion regulation goals, such as maintaining a positive mood or emotional state, improving a negative mood or emotional state, and distracting oneself from worries and unwanted thoughts. The category *celebration* refers to situations such as a birthday, a graduation party, or a wedding. The category *after work* includes situations in which listeners engage with happy music after a busy day at work to celebrate or to relax. The category *motivation* includes situations in which listeners engage with happy music to improve achievement and motivation while coping with a challenging activity such as a job-related task. The category *avoiding happy music* comprises all answers of those participants who reported not to actively select happy music. The category *memory* refers to situations in which happy music is chosen to retrieve autobiographical memories of valued past events or people. The category *musical features* indicates an aesthetic appreciation of happy music focused on the formal properties of the music, rather than on perceptions of emotional content. The number of nominations for the different situational factors is provided in Figure 5. The categories *entertainment*, *celebration, background*, and *mood maintenance* received a total of 196 out of 353 nominations, indicating that participants are especially likely to engage with happy music when with friends or at social occasions, to experience pleasure and enjoyment, and to maintain a positive mood or emotional state. Interestingly, the categories *arousal* and *motor* received a total of 102 out of 353 nominations, indicating that another important use of happy music is to raise or synchronize energy levels, for example, during a morning routine or while physically exercising.

**Table 6:** Summary of the situations in which participants engage with happy music and functions of listening to happy music in those circumstances.

Situation Category	Situation Description	Function
Entertainment	Gathering with friends, social occasions	Social and emotional: use of happy music to entertain, to create a nice atmosphere, and to experience enjoyment
Background	Travelling or while doing aparallel activity such as housekeeping, working, driving	Happy music provides a pleasant background
Motor	Running, dancing, working out	Happy music helps to raise energy level and motivation
Arousal	Energizing in the morning, releasing energy, relaxing	Emotional: arousal and mood regulation
Mood maintenance	Listening to happy music when being in a positive mood or emotional state	Emotional: to maintain a positive mood and to experience enjoyment and pleasure
Celebration	To celebrate (e.g., birthday, graduation, wedding, new year)	Social and emotional: use of happy music to create a nice atmosphere, and to experience enjoyment and pleasure
Mood regulation	Listening to happy music when being in a negative mood or emotional state	Emotional: mood enhancement
After work	After a busy day at work	Happy music is used to relax, celebrate, entertain
Motivation	When copying with a challenging activity	Happy music is used to improve achievement and motivation
Distraction	Listening to happy music toforget about worries andunwanted thoughts	Emotional: diversion or distraction
Avoiding happy music	Preference for other types ofmusic	-
Memory	Retrieving memories ofvalued past events	Happy music as a memory trigger
Musical features	Engaging with happy music not because of its emotionalcontent but rather for the musical features of the piece	Aesthetic

*Note.* Situational categories are listed in descending order according to the number of nominations.

Participants were then asked to rate to what extent they agreed/disagreed with two items, in their ability to describe the liking of happy music: 1) mood-congruent, and 2) mood-incongruent conditions. These questions aimed to explore whether the liking of happy music follows a mood-congruent fashion, as found in the case of sad music. 73.6% of participants (156 out of 212) provided ratings ≥5 on a 7-point Likert scale (1 = *never*, 7 = *always*) in response to the statement “When I am in a positive mood I like to listen to happy music” (M = 5.30, SD = 1.29). On the other hand, only 20.8% of participants (44 out of 212) provided ratings ≥5 on a 7-point Likert scale (1 = *never*, 7 = *always*) in response to the statement “When I am in a negative mood I like to listen to happy music” (M = 3.17, SD = 1.61). A paired-samples t-test revealed a significant difference between the ratings for the two items (*t*(211) = 15.161, *p*<.0001, *r* = .72).

**Figure 6:**
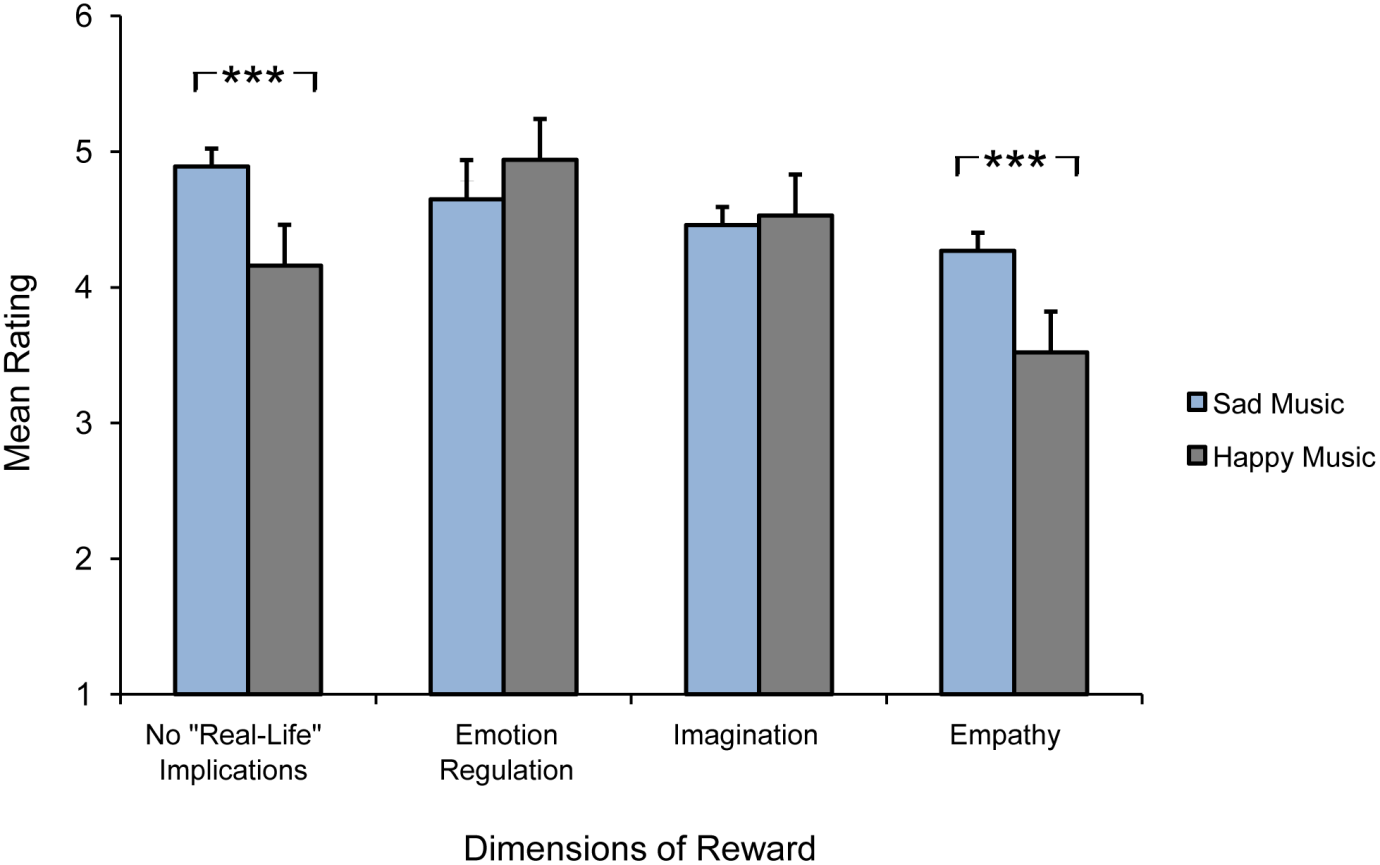
Mean ratings for each of the four dimensions of reward identified for sad and happy music. Error bars indicate standard error of the mean, ***a *p*-level of <.001.

Participants were also asked to complete a questionnaire on the rewarding aspects of music-evoked happiness. The items were the same as those used in the survey on sad music, altered accordingly for happy music. To investigate whether the identified rewards are specific to sad music or also apply to happy music we computed four independent-samples t-tests (one for each reward dimension). This analysis revealed two significant differences. With regard to the *reward of no “real-life” implications*, participants provided higher ratings for sad music (M = 4.89, SE = .05) than for happy music (M = 4.16, SE = .08), with this difference being significant (*t*(982) = 7.476, *p*<.0001, *r* = .23, Bonferroni-corrected). With regard to the *reward of empathy,* participants provided higher ratings for sad music (M = 4.27, SE = .06) than for happy music (M = 3.52, SE = .11), with this difference being significant (*t*(982) = 5.911, *p*<.0001, *r* = .19, Bonferroni-corrected). On the other hand, the *reward of imagination* and the *reward of emotion regulation* did not significantly differ between sad and happy music. Figure 6 shows the mean ratings given to each reward dimension for sad and happy music.

## Discussion

This study dealt with the supposed paradox of why people engage with sad music if sadness is inherently a negative emotion. Using an online survey, we obtained comprehensive responses from a large internet sample. Results point out an extensive confluence between the uses of sad music in everyday life and experiences of reward derived from music-evoked sadness. For example, the use of sad music to regulate negative emotions and moods corresponds to the reward dimension of *emotion regulation*, while the consolatory use is related to the *reward of empathy.* Our findings, which were corroborated by a follow-up survey, are also consistent with previous research suggesting that the principal motivation for listening to sad music is to evoke and influence emotions and moods [57], [58].

### Rewards of music-evoked sadness

With regard to the rewards of music-evoked sadness, a principal components analysis suggests four dimensions, consistent with the possible existence of multiple sources of pleasure, as previously suggested by Huron [59]. Dimension 1 is interpreted as the *reward of imagination* - a dimension that is positively correlated with the pleasure derived from engaging imaginative processes (e.g., imagining to have the same richness and spontaneity of the music). Dimension 2 is interpreted as the *reward of emotion regulation* - a dimension that is positively correlated with the pleasurable outcome derived from the achievement of different self-regulatory goals, such as mood enhancement and venting. Dimension 3 is interpreted as the *reward of empathy* - a dimension that is positively correlated with the pleasurable effects associated with sharing the sadness portrayed by the music as an expression of another’s emotion, such as the composer. This type of pleasure is presumed to relate to social function, even in the absence of other individuals. Dimension 4 is interpreted as the *reward of no “real-life” implications* - a dimension that is positively correlated with a reported pleasure that lacks any extra-musical or contextual implications. In other words, individuals can feel sad in response to sad music even in the absence of extra-musical circumstances that evoke sadness, such as, for example, a lost love (note that this does not necessarily mean that music is not associated to a sad “real-life” situation). Because of this contextual freedom, listeners can take pleasure in music-evoked sadness, by savoring and better understanding its emotional aspects *per se*, without necessarily experiencing negative “real-life” consequences.

### Situational factors and listener characteristics contributing to the appreciation of sad music

The analysis of the situations in which people engage with sad music underlines the importance of the emotional use of sad music (e.g., to regulate negative mood and emotion as well as to take comfort) in everyday life, which is in line with two previous studies investigating the motivations underlying listening to sad music [33], [34]. For instance, our data suggest that people choose to listen to sad music especially when experiencing emotional distress (in most of the cases due to a lost relationship) or when feeling lonely. Correspondingly, participants reported that the liking of sad music is significantly greater when they are sad compared to when they are in a positive emotional state, which is in line with behavioural studies reporting mood-congruent effects on sad music liking [36], [37]. Taken together, these results strongly highlight that, for most of the people, the engagement with sad music in everyday life is correlated with its potential to regulate negative moods and emotions as well as to provide consolation.

The results regarding the contribution of personality traits to individual differences in the appreciation of sad music corroborate findings from previous studies and point out the role of empathy [18]–[20]: Liking of sad music (for both mood-congruent and mood-incongruent conditions) was indeed positively correlated with global *empathy* and its subscale *fantasy*. In addition, we found a negative association between the liking of sad music (when being sad) and the personality trait of *emotional stability*. That is, individuals with high *emotional stability* are less likely to prefer sad music when they are sad. Moreover, the trait *neuroticism* has been linked to the use of music for emotion regulation [52], [60]. Consistent with this, our findings suggest that individuals with low *emotional stability* prefer to listen to sad music when already in a sad state, presumably because this activity can help to regulate their current emotional state. In addition, it could be speculated that individuals with high *emotional stability* are more likely to use happy music to regulate their moods and emotions. If this is indeed the case (our data fall short for a comparison in this regard, since we did not investigate the relationship between personality traits and the appreciation of happy music), the present finding opens an intriguing direction for future work.

### Emotions evoked by sad music

With regard to the emotions evoked in response to sad music, our results reveal that sad music evokes not only sadness, but also a wide range of complex and partially positive emotions, such as nostalgia, peacefulness, tenderness, transcendence, and wonder, in line with a previous study [20]. According to the GEMS model, these emotions belong to the factor of *sublimity*, whereas sadness corresponds to *unease* [23]. In this respect, the present study indicates that the paradox of sad music has largely been discussed in an oversimplified form, based exclusively on the happy-sad dichotomy [61]–[63]. Rather than happiness, sad music elicits an entire range of “sublime” emotions [23]. Moreover, our study supports the observation that music-evoked sadness often occurs in a blended fashion [23]. For instance, the average number of emotions that participants reported to have experienced in response to sad music was above three. Interestingly, the number of emotions evoked by sad music was positively associated with participants’ liking of sad music. This suggests that a multifaceted emotional experience elicited by sad music enhances its aesthetic appeal.

Among the emotions evoked in response to sad music, nostalgia is the one listeners most frequently experience (note that for Eastern participants peacefulness is the most frequent emotion followed by nostalgia). Nostalgia has been characterized as a “bittersweet” emotion because it includes both positive and negative facets simultaneously, such as joy and sadness [64]. A number of studies have stressed the prominence of nostalgia among music-evoked emotions [23], [65], and the present study reinforces the importance of nostalgia in the domain of sad music. The experience of nostalgia also indicates the important role that memory processes play while listening to sad music. Nostalgia is closely linked to the retrieval of autobiographical memories [66], [67]. Barrett and colleagues [64] found that the autobiographical salience of a particular song was the strongest predictor of the intensity of music-evoked nostalgia. In line with this, our analysis of the situations in which participants engage with sad music shows that a motivation to evoke memories of valued past events often underlies the selection of sad music [44], [68].

### Principles underlying music-evoked sadness

Our analysis of the principles underlying emotion evocation points out that *memory* is the most important principle for eliciting sadness. Therefore, the present findings highlight the mediating role of *memory* in the evocation of sadness via music. These results have relevant implications for the experimental design of studies on music-evoked sadness. For example, future experiments could use music-related memory tasks to manipulate sadness in participants. *Contagion* was rated the second most relevant principle after *memory*, and thus also plays an important role in music-evoked sadness. *Emotional contagion* refers to processes where the listener internally mimics the emotional expression of a musical passage [38] in terms of motor expression [69], which is assumed to evoke an emotion due to emotion-specific peripheral physiological feedback. Interestingly, *contagion* was positively correlated with global *empathy* and its subscales, and negatively correlated with *emotional stability*. Studies have shown [70], [71] that emotional contagion is a precursor to empathy, which may explain the positive association between the trait *empathy* and *contagion.* The negative correlation with *emotional stability* suggests that people who are prone to emotional contagion through sad music are also those who have low scores on *emotional stability*. It is noteworthy that global *empathy* correlated with all principles of evocation of sadness, pointing to a strong link between music-evoked sadness and empathy, regardless of the mechanism through which sadness is evoked.

### Uses and rewards of sad music compared to happy music

The results from the follow-up survey on happy music provide some interesting insights into the unique uses and rewards of sad music compared to happy music, which we have summarized in the following three points. *First*, the use of music to regulate negative emotion and mood and to provide comfort is the most relevant use for sad music. This usage, however, appears to be marginal in the case of happy music. For instance, the category *emotion regulation* received only 7.1% of nominations, and the consolatory use of happy music was not mentioned by any participant in the survey on happy music. A number of functions of listening to sad music (i.e., *memory*, *background*, *arousal*) partially overlapped with the functions of happy music, however, the number of nominations they received reveal substantial differences in the significance they hold to listeners when engaging with sad versus happy music. For example, retrieving memories of valued past events is a frequent use of listening to sad music (13.5% of nominations), while it has only marginal relevance in the case of happy music (only 0.9% of nominations). In addition, sad music covers a range of various “inner” functions (directed to one’s own conscious thoughts and feelings) linked to solitary settings (see, for example, the categories of *memory*, *introspection*, and *fantasy*), whereas happy music mainly covers “outer” functions (directed to the sociocultural network to which one belongs) linked to social settings (see, for example, the categories of *entertainment* and *celebration*). *Second*, the liking of happy music follows a mood-congruent pattern, as found for the liking of sad music. This is supported by results from the analysis of the situation-related factors, indicating that listeners frequently engage with happy music to maintain a positive mood or emotional state. Both findings suggest that regulation of negative emotion or mood is a key emotional process underlying the choice to listen to sad music, as is the case for happy music and the maintenance of positive emotion or mood. *Third*, our comparison between the reward questionnaires for sad and happy music indicates that two dimensions of reward, the *reward of no “real-life” implications* and the *reward of empathy,* are rated significantly higher in the case of sad music, suggesting that they represent unique rewarding aspects of music-evoked sadness, in comparison to music-evoked happiness. This appears to be particularly relevant with regard to the *reward of empathy*. For instance, this reward dimension is linked to the consolatory and comforting use of sad music, which was among the most frequently nominated functions of sad music. With regard to the *reward of no “real-life” implications*, our results are consistent with the fact that this type of reward, as conceived by Levinson, applies not to positive emotions, such as happiness, but instead exclusively to negative emotions. On the other hand, no significant difference was found for the *reward of imagination* and the *reward of emotion regulation*, suggesting that these two dimensions of reward may be shared among happy and sad music.

### Cross-cultural differences in the emotional experiences associated with sad music

The survey on sad music featured a large multi-ethnic sample of participants. Therefore, we additionally investigated whether cultural differences affect listeners’ emotional experiences of sad music (i.e., rewarding experiences and principles through which sadness is evoked). We focused on broad cultural differences, namely Western versus Eastern, because a vast and well-established body of literature illustrates an array of West-East differences in psychological processes, including cognition and emotion [72]–[75]. In particular, more individualistic Western cultures are dominated by an independent construal of the self, while more collectivist Eastern cultures are dominated by an interdependent construal of the self [73]. With regard to the principles underlying music-evoked sadness, our results suggest that Western participants experience sadness through memory-related processes more consistently than Eastern participants, in line with Juslin’s [76] theoretical prediction of a high cultural impact on *memory*. Indeed, nostalgia was reported as the most important music-evoked emotion in response to sad music according to Western participants. In contrast, peacefulness holds a parallel significance to nostalgia for Eastern participants. Juslin described that “episodic memories require detached representations, as well as *self-consciousness* (i.e., perceptions of an inner world and a sense of self that is separate from the external world)” (Juslin [76] pp. 242–243), and “episodic memories may serve to confirm one’s identity” (Juslin [77] p. 284). These statements provide a meaningful interpretation of our findings, suggesting that *memory* may serve to promote an independent construal of the self, and is thus more often experienced among individualistic Western cultures. Apart from *memory*, we did not detect any further significant differences, suggesting that the emotional as well as rewarding processes underlying listening to sad music might be largely shared across cultures.

### Listeners’ examples of sad music

Our analysis of the tags given for the respondents’ examples of sad music indicates that a large majority (68.46%) of these musical pieces are also considered sad by last.fm users (notice that among the remaining pieces, 20.80% were assigned a “sadness-related” tag and only 10.74% were not labeled sad). Thus, participants’ view of sad music is consistent with the cultural representation of sadness in music. This finding was confirmed by the analysis of a number of acoustic and musical features, which influence music-perceived sadness in Western music [49]. The average values of tempo, loudness, energy, dance ability, and valence (with the exception of mode) are consistent with the most well-established parameters attributed to Western sad music (in particular, energy, dance ability, and valence values were close to zero, and thus very low in the example pieces named by our sample). Considering the wide range of musical genres covered by the example pieces, the average tempo was relatively slow. Moreover, when energy and valence values for each nominated piece were combined, almost all of the retrievable pieces (113 out of 124) fell into the low energy/negative valence quadrant of the affective circumplex model [51]. According to this dimensional approach, all emotions can be explained in terms of core affect dimensions such as valence and arousal, where sadness is an emotion with low arousal and negative valence [51], [78]. Furthermore, it is noteworthy to mention that our data are in line with the results from the BBC survey on the world’s saddest music [79]. In particular, four out of the five most-nominated pieces of the BBC survey (i.e., *Dido’s Lament*, Barber’s *Adagio for Strings*, *Adagietto* from Mahler’s *Symphony No. 5*, and *Gloomy Sunday*) were also present among the 26 most-rated instrumental pieces in the present study, suggesting that they represent the most popular of the saddest Western musical pieces.

### “Real” and music-evoked sadness

The paradox of the appreciation of music-evoked sadness is closely related to the question of how (and to what extent) music-evoked sadness relates to “everyday” sadness. The fact that sadness is experienced as a pleasant emotion mostly in aesthetic contexts [18], [28] has been used as an argument against the authenticity of music-evoked sadness. Along this line, some authors have argued that music-evoked emotions are not “real”, but *aesthetic* emotions [80], [81], because they are not goal-oriented and do not have any material effect on the individual’s well-being [82], [83]. The present study provides preliminary evidence against the last claim. For instance, two identified rewards (namely *reward of emotion regulation* and *empathy*) unveil two positive effects of music-evoked sadness on psychological health (i.e., regulation of negative emotion and mood, and consolation due to social contact and mood-sharing). This indicates that music-evoked sadness is not only experienced as an abstract aesthetic reward, but also as a means for improving well-being and engaging in social functions. For example, listeners frequently engage with sad music when experiencing emotional distress to facilitate venting of negative emotion or mood. On the other hand, our study also reveals that the lack of “real-life” implications is another reward dimension of music-evoked sadness. According to this reward dimension, music-evoked sadness is often not immediately linked to a sad extra-musical event, thus allowing the listener to take pleasure in so-called negative emotions. This component of music-evoked sadness (the lack of “real-life” implications) differentiates our experience of music-evoked sadness from “everyday” sadness.

### Implications for music therapy

Our findings may have implications for music therapy (MT). For instance, our study shows that music-evoked sadness is linked to four different dimensions of reward, and that sad music can evoke a wide range of positive “sublime” emotions in the listener [23]. Thus, the specific use of sad music in MT (preferably selected by the patient; [84]) might be particularly effective to promote music-induced reward, which in turn could improve health and well-being (through the engagement of neurochemical systems for reward, stress and arousal, as well as social affiliation; for a review, see [85]). This suggestion is supported by the evidence pointing to the efficacy of a form of MT called “Guided Imagery and Music” (GIM) in stress reduction [86], [87]. In healthy subjects, GIM has been shown to reduce cortisol levels [86] as well as β-endorphins [87], which are two markers of hypothalamic-pituitary-adrenal (HPA) axis activations. GIM usually employs Western Classical music combined with conversation and relaxation to elicit imagery for accessing and working through emotional processes. The present study indicates that music-evoked sadness can enhance mental imagery (i.e., *reward of imagination*), thus suggesting that sad music is well suited as one therapeutic means in GIM.

Furthermore, our study reveals that people engage with sad music especially when feeling sad or lonely. Thus, from a therapeutic perspective, one could reasonably interpret a patient’s decision to select sad music as, apart from an aesthetic preference, an indicator of emotional distress. This might be useful especially in children or adults with autism spectrum disorder or alexithymic individuals, who have a reduced ability to express their emotions verbally [88]. By “tuning” their emotions with the ones expressed by the music, patients may feel heard and understood (i.e., *reward of empathy*), even in the absence of a specific emotional vocabulary [89]. This empathic connection between the music and the patient may help to relieve distress and to progress in therapy. Furthermore, the beneficial emotional effects of sad music may be enhanced in emotionally unstable individuals, because our results suggest that they use sad music to regulate emotion. Thus, we also propose that the assessment of personality traits might be an important stage in estimating the successful use of sad music in MT.

### Limitations of the study

The use of a retrospective survey may have limited the overall ecological validity of the study. Retrospective questionnaires can be inaccurate in the measurements of affective states [90], because they are vulnerable to memory biases [91], [92]. Future research investigating everyday use of sad music could potentially benefit from the Experience Sampling Method (ESM) [93], a research procedure that asks individuals to provide systematic self-reports of their experience in real-time and at random occasions during the day. In particular, a recent development of ESM, consisting of a “smartphone” application (m-ESM) [90], seems to be a promising solution because it maintains a natural listening experience for participants while collecting real-time data. In addition, further studies should validate the present findings with implicit measures to overcome the limitations of introspective survey methods, such as demand characteristics. This is important especially in regard to the rewards and principles underlying music-evoked sadness, which describe processes that may occur partly unconsciously, thereby proving more difficult for direct reporting by participants.

## Conclusions

The fact that people seek and appreciate sadness in music may appear paradoxical, given the strong popular and scientific emphasis on happiness as a source of personal well-being (e.g., [94], [95]). The present study demonstrates that for many individuals, listening to sad music can actually lead to beneficial emotional effects. Our findings are important for four reasons. *First*, the findings (using two large internet samples of participants) reveal sad music’s potential for regulating negative moods and emotions as well as for providing consolation. In particular, the consolatory and comforting effects are likely to be unique features of sad music, as suggested by the comparison between the uses and functions of listening to sad versus happy music. *Second*, the results draw a comprehensive picture of situational factors of exposure and personality traits that contribute to the appreciation of sad music. In particular, the appreciation of sad music is enhanced when listeners are experiencing emotional distress, as well as among individuals with high empathy and low emotional stability. *Third*, our results unveil psychological mechanisms underlying the evocation of sadness by music, showing that memory-related processes are central in music-evoked sadness. *Fourth*, our findings contribute to the discussion surrounding the paradox of music-evoked sadness by providing the first empirical evidence that music-evoked sadness is related to a multidimensional experience of reward: Music-evoked sadness can be appreciated not only as an aesthetic abstract reward (due to the engagement of imaginative processes or the lack of “real-life” implications), but also plays a role in well-being, by providing consolation as well as by regulating negative moods and emotions. In particular, the results from the follow-up survey on happy music suggest that two out of the four identified rewards, the *reward of no “real-life” implications* and the *reward of empathy*, are rewarding experiences derived from listening to sad music, but not happy music (although rewarding experiences derived from listening to other types of music remain to be specified in future research). We hope that this study will lead to a deeper understanding of music-evoked sadness and will spur further research into the relationship between sadness and pleasure, particularly in the domain of music-therapeutic applications. Potential implications include the development of music interventions designed to improve health and well-being in healthy subjects as well as in the treatment of psychiatric disorders.

## Supporting Information

Figure S1 **Scatter plot of valence and energy values of the retrieved instrumental musical pieces.**
(PDF)

Table S1 **List of items of the questionnaire on sad music listening habits (third section of the survey).**
(PDF)

Table S2 **List of items of the questionnaire on the principles underlying the evocation of sadness by music (fourth section of the survey).**
(PDF)

Table S3 **List of items of the questionnaire on the rewarding aspects of music-evoked sadness (fifth section of the survey).**
(PDF)

Table S4 **Free responses to the item asking which are the rewarding aspects of sadness evoked by music, with number of nominations for each answer (N = 31 in total).**
(PDF)

Table S5 **Free responses to the item asking which are the most frequent emotions experienced in response to sad music, with number of nominations for each answer (N = 40 in total).**
(PDF)

Table S6 **Respondents’ demographics for the happy music survey (N = 212).**
(PDF)

Table S7 **Musical pieces nominated more than one time.**
(PDF)

Table S8 **Musical pieces nominated one time.**
(PDF)

Dataset S1 **Original dataset collected though an online platform for the sad music survey.**
(XLSX)
