# Supplementary material for: The Paradox of Music-Evoked Sadness: An Online Survey
Source: PLoS One. 2014 Oct 20;9(10):e110490. doi: 10.1371/journal.pone.0110490 (PMC4203803; doi:10.1371/journal.pone.0110490)

Figure S1. Scatter plot of valence and energy values of the retrieved instrumental musical pieces. Valence values ranged between 0 and 1. A value close to 1 indicates a positive emotion, while a value close to 0 is a negative emotion. Energy values ranges between 0 and 1. A value close to 1 indicates high energy or arousal, while a value close to 0 corresponds to low energy or arousal. The dotted lines divide the area into the 4 quadrants of the circumplex model of emotion: high energy/positive valence, high energy/negative valence, low energy/positive valence, and low energy/negative valence. According to this model, sadness corresponds to the low energy/negative valence quadrant.

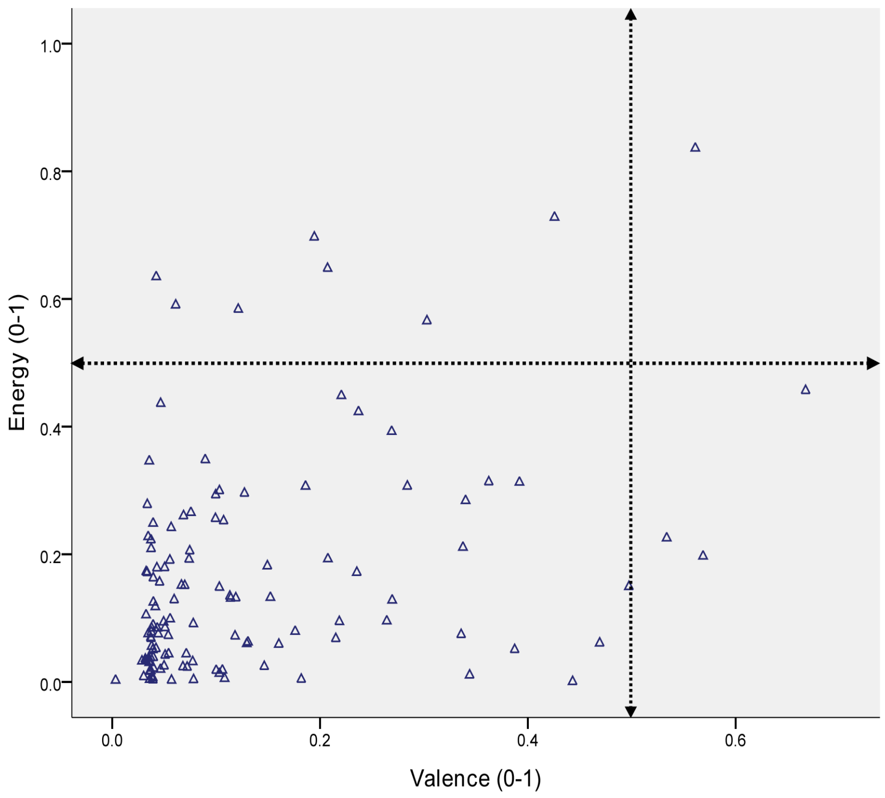

Supplement: Figure S1 — Scatter plot of valence and energy values of the retrieved instrumental musical pieces. (PDF) [file pone.0110490.s001.pdf]
