# Supplementary material for: The Paradox of Music-Evoked Sadness: An Online Survey
Source: PLoS One. 2014 Oct 20;9(10):e110490. doi: 10.1371/journal.pone.0110490 (PMC4203803; doi:10.1371/journal.pone.0110490)
Supplement: Table S1 — List of items of the questionnaire on sad music listening habits (third section of the survey). (PDF) [file pone.0110490.s002.pdf]

Table S1. List of items of the questionnaire on sad music listening habits (third section of the survey).

Item	Item Text
Frequency of listening to sad music	How often do you actively select sad music to listen to?
Importance of situation-related factors	How much do specific situations influence your choice to listen to sad music?
Example of situation-related factors	Could you write down one or more examples of situations in which you engage with sad music and why?
Mood-congruent liking of sad music	When I am in a sad mood I like to listen to sad music.
Mood-incongruent liking of sad music	When I am in a positive mood I like to listen to sad music.
Emotions evoked by sad music: Sadness	Sad music makes me feel sad or sorrowful.
Tenderness	Sad music makes me feel tender, affectionate or in love.
Nostalgia	Sad music makes me feel nostalgic, dreamy or melancholic.
Tension	Sad music makes me feel tense, agitated or nervous.
Peacefulness	Sad music makes me feel serene, calm or soothed.
Joyful Activation	Sad music makes me feel joyful, amused or bouncy.
Transcendence	Sad music makes me feel fascinated or overwhelmed and evokes in me feelings of transcendence or spirituality.
Wonder	Sad music makes me feel filled with wonder, dazzled or moved.
Power	Sad music makes me feel strong, triumphant or energetic.
Any emotion	Sad music does not evoke in me any particular emotion.
