# Supplementary material for: The Paradox of Music-Evoked Sadness: An Online Survey
Source: PLoS One. 2014 Oct 20;9(10):e110490. doi: 10.1371/journal.pone.0110490 (PMC4203803; doi:10.1371/journal.pone.0110490)
Supplement: Table S2 — List of items of the questionnaire on the principles underlying the evocation of sadness by music (fourth section of the survey). (PDF) [file pone.0110490.s003.pdf]

Table S2. List of items of the questionnaire on the principles underlying the evocation of sadness by music (fourth section of the survey).

Principle	Item Text
Memory	Sad music makes me feel sad because it evokes memories of certain past events, people or places.
Contagion 1	I am affected by the expression of sadness in music to the point that I frown or even cry.
Contagion 2	Sad music makes me feel sad because its musical features (e.g., slow tempo, legato articulation, etc.) evoke a sad mood in myself.
Appraisal 1	Sad music helps me to be sad when I want to be sad.
Appraisal 2	Sad music can evoke sad mood when it is appropriate (e.g., funeral).
Imagination	Sad music makes me feel sad because I imagine sad objects/scenes.
Social Functions	Sad music makes me feel sad because I am touched by the sadness of others.
