# Supplementary material for: The Paradox of Music-Evoked Sadness: An Online Survey
Source: PLoS One. 2014 Oct 20;9(10):e110490. doi: 10.1371/journal.pone.0110490 (PMC4203803; doi:10.1371/journal.pone.0110490)
Supplement: Table S3 — List of items of the questionnaire on the rewarding aspects of music-evoked sadness (fifth section of the survey). (PDF) [file pone.0110490.s004.pdf]

Table S3. List of items of the questionnaire on the rewarding aspects of music-evoked sadness (fifth section of the survey).

Item Name	Item Text (I like to listen to sad music because...)
Understanding Feelings	... by contemplating this feeling in the music, I can get a better understanding of my own feelings, without negative life consequences.
Emotional Assurance	... I can reassure myself - in a non-destructive manner - of the depth of my ability to feel.
Savoring Feeling	... I can enjoy the pure feeling of sadness in a balanced fashion, neither too violent, nor as intense as in real-life.
Mood Enhancement	... sad music helps me to regulate my mood (e.g. feel better) when I am feeling sad.
Catharsis/Venting	... experiencing sadness through music makes me feel better after listening to it, and thus has a positive impact on my emotional well-being.
Expressive Potency 1	... I imagine I have the same RICH expressive ability as present in the music.
Expressive Potency 2	... I imagine I have the same POWERFUL expressive ability as present in the music.
Expressive Potency 3	... I imagine I have the same SPONTANEOUS expressive ability as present in the music.
Emotional Communion	... I imagine I share the same emotional experience of the composer and thus I do not feel alone anymore.
Empathic Responses	... I like to empathise with the sadness expressed in the music, as if it were another individual.
Emotional Resolution	… I can match my emotional state to the one that unfolds over the course of the music, maintaining at the same time a sense of control over it.
Realistic Thinking	… it makes me think more realistically about life.
Apprehending Expression	… it facilitates my grasp of the musical work.
