# Supplementary material for: The Paradox of Music-Evoked Sadness: An Online Survey
Source: PLoS One. 2014 Oct 20;9(10):e110490. doi: 10.1371/journal.pone.0110490 (PMC4203803; doi:10.1371/journal.pone.0110490)
Supplement: Table S4 — Free responses to the item asking which are the rewarding aspects of sadness evoked by music, with number of nominations for each answer (N = 31 in total). (PDF) [file pone.0110490.s005.pdf]

Table S4. Free responses to the item asking which are the rewarding aspects of sadness evoked by music, with number of nominations for each answer (N=31 in total). Note that similar answers are grouped together.

**Rewarding aspects of music-evoked sadness**	**N. Nominations**
Sad music calms me down or relaxes me.	10
Sad music helps me to release feelings of sadness or loss.	9
Sad music evokes intense emotions or makes me feel alive.	4
Sad music connects me to the suffering of others and makes me feel less alone.	4
Sadness is expressed non-violently and it is enjoyable because music is a safe environment.	2
Sad music helps me to maintain motivation during long working periods.	2
