# Supplementary material for: The Paradox of Music-Evoked Sadness: An Online Survey
Source: PLoS One. 2014 Oct 20;9(10):e110490. doi: 10.1371/journal.pone.0110490 (PMC4203803; doi:10.1371/journal.pone.0110490)
Supplement: Table S5 — Free responses to the item asking which are the most frequent emotions experienced in response to sad music, with number of nominations for each answer (N = 40 in total). (PDF) [file pone.0110490.s006.pdf]

Table S5. Free responses to the item asking which are the most frequent emotions experienced in response to sad music, with number of nominations for each answer (N=40 in total). Note that similar answers are grouped together.

Emotions experienced in response to sad music	N. Nominations
Cathartic experience or feeling of relief	12
Feeling of being understood and connected with others	10
Feeling of being moved	8
Thoughtful or introspective	4
Hopeless or weak	2
Regretful	1
Creative	1
Romantic	1
Melodramatic	1
