# Supplementary material for: The Paradox of Music-Evoked Sadness: An Online Survey
Source: PLoS One. 2014 Oct 20;9(10):e110490. doi: 10.1371/journal.pone.0110490 (PMC4203803; doi:10.1371/journal.pone.0110490)
Supplement: Table S6 — Respondents’ demographics for the happy music survey (N = 212). (PDF) [file pone.0110490.s007.pdf]

Table S6. Respondents' demographics for the happy music survey (N=212).

	Number of respondents	Percentage of respondents
**Gender**		
**Male**	75	35.4
**Female**	137	64.6
**Musical training**		
**professional musicians**	22 (7 females)	10.4
**semi-professional musicians**	27 (11 females)	12.7
**amateur musicians**	76 (51 females)	35.8
**non-musicians**	87 (68 females)	41
**Place of origin**		
**Africa**	1 (1 female)	0.5
**Asia**	41 (26 females)	19.4
**Australia**	2 (1 female)	1
**Europe**	148 (98 females)	69.7
**North America**	12 (6 females)	5.6
**South America**	8 (5 females)	3.8
