# Supplementary material for: The Paradox of Music-Evoked Sadness: An Online Survey
Source: PLoS One. 2014 Oct 20;9(10):e110490. doi: 10.1371/journal.pone.0110490 (PMC4203803; doi:10.1371/journal.pone.0110490)
Supplement: Table S7 — Musical pieces nominated more than one time. (PDF) [file pone.0110490.s008.pdf]

Table S7. Musical pieces nominated more than one time.

Title	Composer	Number of Nominations	Tags
Adagio for strings Op.11	Samuel Barber	9	sad
Adagio in G minor	Tomaso Albinoni	7	sad
Address in the stars	Caitlin & Will	2	not many tags
Air on the 4th string: Suite No. 3 in D major, BWV 1068	Johann Sebastian Bach	2	sad
Ashes in the snow	Mono	2	melancholic
Bedshaped	Keane	2	sad
Cello suite No. 1 (Prelude)	Johann Sebastian Bach	2	melancholic
Colorblind	Counting Crows	2	sad
Comfortably numb	Pink Floyd	2	sad
Dance with my father	Luther Vandross	2	sad
Dido's lament	Henry Purcell	3	dramatic, tragic
Exit music (for a film)	Radiohead	3	sad
Fade to black	Metallica	2	sad
Fix you	Coldplay	2	sad
Für Elise: Bagatelle No. 25 in A minor	Ludwig van Beethoven	2	sad
Gloomy Sunday: The suicide song	Rezso Seress	2	melancholic
Gnossienne No.1	Erik Satie	2	sad
Gymnopédie No. 1	Erik Satie	6	sad
Hallelujah	Leonard Cohen/Jeff Buckley (cover)	2	sad
Hurt	Johnny Cash	6	sad
Il cantico dei drogati	Fabrizio De Andre'	2	not found
In a sentimental mood	Duke Ellington	2	sad
Kindertotenlieder	Gustav Mahler	2	sad
Kol Nidrei Op. 47	Max Bruch	2	rainy day, bittersweet blues
Lonely day	System of a Down	3	sad
Lux aeterna (Requiem for a dream OST)	Clint Mansell	2	sad
Mad world	Gary Jules	3	sad
Maggot brain	Funkadelic	2	sad
Mass in B minor BWV 232	Johann Sebastian Bach	2	not many tags
Moon reflected in the second spring	Ah Bing	8	not found
Moonlight sonata: Piano sonata No. 14 in C-sharp minor, Op. 27, No. 2	Ludwig van Beethoven	12	sad
My hearth will go on	Celine Dion	2	sad
Nocturnes	Frederic Chopin	3	melancholic
Pathétique: Symphony No. 6 in B minor, Op. 74	Pyotr Ilyich Tchaikovsky	2	classical, russian
Piano concerto No. 2 in C minor, Op. 18	Sergei Rachmaninoff	2	mellow
Requiem Mass in D minor (K. 626)	Wolfgang Amadeus Mozart	5	sad
River flows in you	Yiruma	3	sad
Skinny love	Bon Iver	2	sad
Someone like you	Adele	5	sad
Street spirit (fade out)	Radiohead	2	sad

Symphony No. 5 (Adagietto)	Gustav Mahler	3	sad
Symphony of sorrowful songs: No. 3, Op. 36	Henryk Górecki	3	sorrow
Symphony No. 7 in A major, Op. 92 (Allegretto)	Ludwig van Beethoven	3	sad
Tears in heaven	Eric Clapton	4	sad
The heart asks pleasure first (The piano OST)	Michael Nyman	5	melancholy
The Planets Op. 32: Jupiter	Gustav Holst	2	melancholic, more sad than jolly
The tourist	Radiohead	2	sad
Theme from Schindler's list	John Williams	5	sad
This woman's work	Kate Bush	2	sad
Tristan und Isolde (Prelude)	Richard Wagner	3	any tag
Yesterday	Beatles	3	sad
You are my sister	Antony and the Johnsons	2	sad
