# Supplementary material for: The Paradox of Music-Evoked Sadness: An Online Survey
Source: PLoS One. 2014 Oct 20;9(10):e110490. doi: 10.1371/journal.pone.0110490 (PMC4203803; doi:10.1371/journal.pone.0110490)
Supplement: Table S8 — Musical pieces nominated one time. (PDF) [file pone.0110490.s009.pdf]

Table S8. Musical pieces nominated one time.

Title	Composer/Singer	Tags
10,000 miles	Mary Chapin Carpenter	sad
A case of you	Tori Amos	sad
A falling through	Ray Lamontagne	sad, sadness
A vava inouva	Idir	nostalgia
Abyss	Sakamoto & Fennesz	sad
Afraid not scared	Ryan Adams	sad
Again	Archive	sad
Al respirar	Vetusta morla	indie, spanish
All by myself	Eric Carmen	sad
All I wanted	Paramore	depressing
And I walk with them	My Dying Bride	sorrow
Angels	The XX	melancholic
Angkor Wat – Theme Finale (I n the mood for love OST)	Michael Galasso	sad
Any other time (American beauty OST)	Thomas Newman	sad
Après un rêve Op. 7 No. 1	Gabriel Fauré	sad
Ara batur	Sigur Ros	sad
Are you there?	Mono	sad
As tears go by	Rolling Stones	sad
Ave Maria	Franz Schubert	sad
Avril 14th	Aphex Twin	sad
Barcarolle	Jacques Offenbach	sad
Because of you	Kelly Clarkson	sad
Beneath your beautiful	Labrinth feat. Emeli Sandé	beautiful, pop
Better off dead	Anathema	sad
Between the bars	Elliott Smith	sad
Bide my Time	Samiam	guitar, melodic
Blindness	Metric	melancholic
Blue calx	Aphex Twin	dreamy, peaceful
Born to die	Lana Del Rey	sad
Breathless	The Corrs	pop, romantic
Brick	Ben Folds	sad
Butterflies instead	K's Choice	melancholic
Bye bye baby	Bay City Rollers	pop, oldies
Canon in D Major	Johann Pachelbel	mellow
Cantus in memory of Benjamin Britten	Arvo Pärt	sad
Canzone per un'amica	Guccini	folk, italian
Careless whisper	George Michael	sad
Cavalleria rusticana (Intermezzo)	Pietro Mascagni	sad
Children	Robert Miles	sad
Ci sono molti modi	Afterhours	sad
Cinema paradiso theme	Ennio Morricone	sad
Clair de lune	Claude Debussy	melancholic
Comptine d'un autre été l'après-midi (Amelie OST)	Yann Tiersen	sad
Con te partiro'	Andrea Bocelli	melancholy

Cry	Kelly Clarkson	sad
Cry me a river	Diana Krall	mellow
Dark was the night, cold was the ground	Blind Willie Johnson	melancholic
Dead indeed	Nils Petter Molvaer	drum and bass, jazz
Dear agony	Breaking Benjamin	sad
Death of Evita (Evita OST)	Andrew Lloyd Webber	sad
Deborah's theme (Once upon a time in America OST)	Ennio Morricone	sad
December song	George Michael	christmas, pop
Die Moldau (Vltava)	Smetana	mellow
Dirge	Death In Vegas	melancholic
Disintegration	The Cure	sad
Don't take the girl	Tim McGraw	sad
Drown out	Glen Hansard & Marketa Irglowa	saddest
Dying	Hole	sad
East to west	Casting Crowns	praise, worship
Everybody hurts	REM	sad
Eyala	Richard Bona	melancholic
Far Away	Jay Jay Johanson	sad
Father & son	Cat Stevens	melancholy
Feel something	Holy Other	downtempo, dub
Feeling good	Nina Simone	nostalgia
For today I am a boy	Antony and the Johnsons	sad
Für Alina	Arvo Pärt	sad
Full of grace	Sarah McLachlan	sad
Funny time of the years	Beth Gibbons & Rustin Man	sad
Gabriel's oboe (The Mission OST)	Ennio Morricone	mellow
Generique	Miles Davis	sad
Ghost song	Patrick Wolf	Indie, romantic and dark
Giorni dispari	Ludovico Einaudi	sad
Golden age	Beck	sad
Gollum's song (The Lord of the rings OST)	Howard Shore	sad
Gone too soon	Simple Plan	sad
Good night	The Beatles	melancholy
Graduation	Vitamin C	sad
Grey room	Damien Rice	sad
Hallowed be thy name	Iron Maiden	heavy metal, metal
Hello, I'm in Delaware	Dallas Green	sad but wonderful
Help yourself	Sad Brad Smith	sad
Highschool lover	Air	melancholic
Home	Foo Fighters	sad
Home	Michael Buble	sad
Hope there's someone	Antony and the Johnsons	sad
How can you mend a broken hearth	Al Green	sad
How do you get that lonely	Blaine Larsen	sad
How it ends	DeVotchKa	sad
How to disappear completely	Radiohead	sad

Hurt	Christina Aguilera	sad
I don't blame you	Cat Power	sad
I giorni	Ludovico Einaudi	calm, peaceful
I grieve	Peter Gabriel	sad
I will follow you in the dark	Death Cab For Cutie	sad
I will remember you	Sarah McLachlan	sad
I wish it would rain down	Phil Collins	sad
I'm so happy I can't stop crying	Sting	sad
In praise of dreams	Jan Garbarek	evening, night
Infra 5	Max Richter	sad
Iris	Wim Mertens	melancholy
Ironic	Alanis Morissette	sad
Isle of the dead Op.29	Sergei Rachmaninoff	classical, rach
Jamie all over	Mayday Parade	alternative, emo
Jazz suite waltz No.2	Dmtri Shostakovich	russian, waltz
Joga	Björk	melancholy
Julia	Pavlov's Dog	mellow
Junimond	Rio Reiser	sad
Kashmir	Led Zeppelin	dramatic
Kiss the rain	Yiruma	sad
Kissing you	Des'ree	sad
Längtar bort från mitt hjärta	Shining	sad
Last date	Floyd Cramer	oldies, soothing
Le onde	Ludovico Einaudi	melancholic
Learning	Perfume Genius	melancholy
Leaving on a jet plane	John Denver	sad
Legends of the fall OST	James Horner	drama, silence
Les passantes	Georges Brassens	melancholy
Let her cry	Hootie & the Blowfish	sad
Letters to the metro	Mogwai	depressing
Letting go	Isaac Shepard	piano, relax
Like a sad song	John Denver	sad
Lithium	Nirvana	sad
Ljósið	Ólafur Arnalds	melancholic
Lord kill the pain	Red House Painters	sadcore
Losing you	Solange	heartbreak
Lover, you should've come over	Jeff Buckley	sad
Lucky	Radiohead	sad
Lullaby	Nickelback	sad
Main title (Bravehearth OST)	James Horner	sad
Majesty	Madrugata	melancholy
Mama hold my hand	Aloe Blacc	sad
Maybe not	Cat Power	sad
Memory (Cats Musical)	Andrew Lloyd Webber	sad
Merry Christmas, Mr. Lawrence	Ryuichi Sakamoto	sad
Miserere mei, Deus	Gregorio Allegri	melancholy
Mistral gagnant	Renaud	sad
Monday	Ludovico Einaudi	sad
Mother	John Lennon	sad

Moya	Godspeed You! Black Emperor	melancholic
My December	Linkin Park	sad
My immortal	Evanescence	sad
My Insect Life	Kaki King	mellow
Nights in white satin	Moody Blues	sad
Nimrod variation IX (from Enigma variations Op.36)	Sir Ewdard Elgar	sad
No more 'I love you's'	Annie Lennox	sad
No need to argue	The Cramberries	sad
Nostalgia	Yanni	nostalgia
Nothing compares to you	Sinéad O’Connor	sad
November rain	Guns N' Roses	sad
Now You're Taken	Mogwai	sad
Numbing the pain	Heaven Shall Burn	depressing
Nur zu besuch	Die Toten Hosen	sad
Ohne dich	Selig	sad
On the Nature of daylight	Max Richter	sad
Out from Under	Britney Spears	sad
Overnight	Gonzales	sad
Pavane Op.50	Gabriel Faure	melancholic
Pavane pour une infante defunte	Maurice Ravel	sad
Piano sonata No. 11 in A major K 331 Alla Turca (Andante Grazioso)	Mozart	mozart, piano
Possibility	Lykke Li	sad
Promise me	Beverley Craven	sad
Redemption song	Bob Marley	sad
Remembrance day	God Is An Astronaut	melancholy
Rhapsody on a theme of Paganini, Op. 43	Sergei Rachmaninoff	piano, romantic
Ride	Lana Del Rey	sad
Road Trippin'	Red Hot Chili Peppers	sad
Rootless tree	Damien Rice	sad
Roulette	System of a Down	sad
Safe and sound	Taylor Swift	sad
Sam Stone	John Prine	sad
Santa monica	Theory of a Deadman	sad
Say it's not true	Queen	heartbreaking
Say my name	Within Temptation	sad
Second skin	The Chameleons	melancholy
Selective Memory	Eels	sad
Shadow of the day	Linkin Park	sad
Sheherazade (Overture)	Rimsky-Korsakov	classical, romantic
Shine on you crazy diamond	Pink Floyd	melancholy
Siempre Me Quedara	Bebe	sad
Silver spring	Fleetwood Mac	sad
Sodade	Cesaria Evora	sad
Soldier on	The Temper Trap	sad
Sometimes	City & Colour	sad
Song from a secret garden	Secret Garden	sad
Sorry seems to be the hardest world	Elton John	sad

Space-dye vest	Dream Theater	sad
Spiegel im spiegel	Arvo Pärt	sad
Stabat Mater dolorosa in F minor RV 621	Antonio Vivaldi	really sad
Stay	Rihanna	sad
Still I'm sad	The Yardbirds	sad
Strange fruit	Billie Holiday	sad
Supermassive black hole	Muse	alternative, rock
Superstar	Sonic Youth	sad
Symphony No.3 Op. 90 (3rd movement – Poco Allegretto)	Johannes Brahms	sad
Symphony No.9, Largo (from The new world Op.95)	Antonín Dvořák	sad
Teardrop	Massive Attack	sad
Terrible lie	Tool	sad
The bad in each other	Feist	canadian, indie
The blower´s daughter	Damien Rice	sad
The chairman's waltz (Memories of a geisha OST)	John Williams	sad
The colour of spring	Mark Hollis	sad
The cry of mankind	My Dying Bride	sad
The end	The Doors	sad
The funeral march: Piano sonata No. 2 in B-flat minor, Op. 35	Frédéric Chopin	sad
The ghosts you draw on my back	Mum	melancholy
The ice is getting thinner	Death Cab For Cutie	sad
The Journey	Boston	melodic rock, soft rock
The last man (The fountain OST)	Clint Mansell	sad
The letter that never came	Thomas Newman	sad
The lonely shepherd	James Last	sad
The love - Lily's theme (Harry Potter OST)	Alexander Desplat	sad
The messiah will come again	Roy Buchanan	makes me cry
The music of the night	Andrew Lloyd Webber	sad
The Only Exception	Paramore	sad
The race is on	George Jones	country, love songs
The Rifle	Alela Diane	mellow
The rose	Conway Twitty	country, love songs
The sound of silence	Simon & Garfunkel	sad
The tide	The Spill Canvas	sad
The way she feels	Between The Trees	sad
The weeping song	Nick Cave & the Bad Seeds	sad
Thieves	She & Him	nostalgia
Time lapse	Nyman	melancholy
Too many humans	Buckethead	melancholic
Trilogy (The last chapter)	ATB	nostalgia
True love waits	Radiohead	sad
Truman sleeps (Truman Show OST)	Philip Glass	sad
Unbreak my heart	Toni Braxton (Celine Dion's cover)	sad

Undenied	Portishead	sad
Untitled #3	Sigur Ros	nostalgic
Vaka	Sigur Ros	sad
Violin concerto in E minor Op.64 (I mov)	Felix Mendelssohn	melancholy
Vocalise Op.34 No.14	Sergei Rachmaninoff	melancholy
Volver	Estrella morente	melancholy
Walking in my shoes	Depeche Mode	sad
Waterfront	The Black Hearth Procession	depression
Watermark	Enya	calm, new age
Weather storm	Craig Armstrong	sad
What I cannot change	LeAnn Rimes	depression
What if I do	Foo Fighters	sad
When I was your man	Bruno Mars	sad
Whisky lullaby	Brad Paisley	sad
Who you'd be today	Kenny Chesney	sad
Why	Lennox	sad
Why does my heart feel so bad	Moby	sad
Wire to wire	Razorlight	sad
Wish you were here	Pink Floyd	sad
With or without you	U2	sad
Words	Low	sad
You belong to me	Bob Dylan	sad sad song
